# Clinical and CSF Measurements for Identification of Leptomeningeal Metastasis in Lung Adenocarcinoma

**DOI:** 10.3390/diagnostics16172742

**Published:** 2026-08-27

**Authors:** Qichao Wu, Beihe Cao, Lei Qiu, Juan An, Yutong Zheng, Yufeng Shi, Xiaoyan Li

**Affiliations:** Department of Oncology, Beijing Tiantan Hospital, Capital Medical University, No. 119 South 4th Ring West Road, Fengtai District, Beijing 100070, China; wuqichao0529@163.com (Q.W.); caobeihe0509@163.com (B.C.); qlshjtdx@163.com (L.Q.); angel0311@163.com (J.A.); yutongzheng48@gmail.com (Y.Z.);

**Keywords:** leptomeningeal metastasis, lung adenocarcinoma, cerebrospinal fluid, diagnostic model, internal evaluation, nested cross-validation, brain metastasis

## Abstract

**Background/Objectives:** Because brain MRI and initial CSF cytology have limited sensitivity, suspected leptomeningeal metastasis (LM) may remain unresolved. We developed a model using clinical and CSF measurements available in source records to support identification of current LM in patients with lung adenocarcinoma undergoing CSF evaluation. **Methods:** This single-center retrospective cohort included 533 patients with lung adenocarcinoma and CNS metastases: 366 with a retrospectively verified reference diagnosis of current LM and 167 with brain metastases only. An eight-variable logistic model underwent repeated nested cross-validation. Conditional internal performance was estimated using outer-split thresholds selected by inner resampling at specificity >0.90. **Results:** Mean outer held-out AUROC was 0.960 (95% CI, 0.942–0.974), Brier score was 0.068 (0.054–0.084), calibration slope was 0.864 (0.708–1.106), sensitivity was 0.912 (0.883–0.938), and specificity was 0.901 (0.857–0.939). In a descriptive full-cohort analysis, the model flagged 157 of 170 (92.4%) current-LM patients not identified by the brain MRI strategy and 59 of 65 (90.8%) not identified by first CSF cytology for further evaluation. These were not independent head-to-head accuracy comparisons because MRI and cytology contributed to the reference diagnosis. **Conclusions:** In this selected cohort, the clinical and CSF measurements captured a multivariable pattern associated with a retrospectively verified reference diagnosis of current LM. The model showed strong conditional internal performance and may serve as an investigational adjunct to prompt further LM-directed evaluation. Independent multicenter validation is required before clinical use.

## 1. Introduction

Leptomeningeal metastasis (LM) is a clinically consequential pattern of central nervous system (CNS) progression in non-small cell lung cancer, particularly in lung adenocarcinoma. Longer survival and CNS-active therapies have increased the need to define the site and pattern of intracranial disease [1,2]. LM can coexist with parenchymal brain metastases, emerge after earlier CNS treatment, and present with nonspecific neurologic symptoms. Its observed epidemiology therefore depends on referral, surveillance, and competing CNS disease patterns [3]. In patients with established CNS metastatic involvement, the immediate question is whether disease includes the leptomeningeal compartment rather than parenchymal metastases alone. This distinction can alter the diagnostic workup, interpretation of neurologic symptoms, treatment planning, and follow-up intensity. Failure to recognize LM may delay reassessment, whereas unnecessary alerts may lead to additional procedures and patient burden. This problem is concentrated among patients referred for lumbar puncture because LM is already suspected. They constitute a selected diagnostic population, not a general screening population for lung adenocarcinoma. A useful quantitative tool must address uncertainty within this pathway without being interpreted as applicable to all patients with lung cancer or brain metastases.

Current LM diagnosis is appropriately based on integrated clinical evaluation, contrast-enhanced magnetic resonance imaging (MRI), cerebrospinal fluid (CSF) cytology, and disease context, rather than a single universally sufficient test [4,5]. Positive cytology provides direct evidence of malignant cells, but a negative first sample does not exclude LM; optimized collection and processing and, when clinically indicated, repeat sampling remain important [4]. MRI supplies essential anatomical information, yet enhancement may be absent, subtle, treatment related, or interpreted variably; dedicated scorecard studies have therefore emphasized standardized acquisition and interpretation [6]. A meta-analysis of 10 studies involving 668 patients with solid cancers quantified these limitations [7]. Pooled sensitivity was 71.9% (95% CI, 54.7–82.9%) for first CSF cytology and 59.4% for contrast-enhanced MRI, although MRI retained high pooled specificity at 97.6% (95% CI, 77.3–99.8%). Cytology specificity could not be independently estimated with confidence because positive cytology frequently contributed to the reference diagnosis. The two modalities assess different disease domains: cytology samples shedding of malignant cells at a single lumbar puncture, whereas MRI depicts anatomical abnormalities at one imaging time point. Neither modality alone is sufficiently sensitive to exclude LM after a negative initial result. These false-negative results matter because LM may remain unrecognized, necessitating repeat CSF sampling or serial imaging and potentially delaying diagnostic confirmation and LM-directed management [4,7,8]. When suspicion persists despite negative or equivocal routine evidence, clinicians therefore need an additional signal to guide further LM-directed evaluation while leaving the final diagnostic determination with the treating team.

CSF measurements obtained during clinical evaluation may provide complementary information because they quantify tumor marker, cellular, and biochemical changes in the sampled compartment. CSF CEA and CYFRA21-1 have shown adjunctive diagnostic value in LM, including in lung cancer cohorts, while WBC, glucose, protein, lactate, and chloride frequently vary with malignant CSF involvement [9,10,11,12]. Unlike a binary cytology result, these variables are continuous and potentially repeatable; however, availability varies across centers, particularly for CSF CEA and CYFRA21-1. Existing multivariable studies have examined mixed solid tumors, clinicopathologic nomograms for lung adenocarcinoma, and tumor marker panels for NSCLC [12,13,14]. CTC, cell-free DNA, and metabolomic studies also show that LM can leave high-dimensional signals in CSF, but these technologies require specialized platforms and address partly different diagnostic or molecular questions [15,16,17]. Few studies have integrated CSF tumor markers with cellular, biochemical, and clinical context measurements in patients with lung adenocarcinoma and CNS metastases undergoing CSF evaluation. Evidence is also limited for the model-development and internal-evaluation pathway, including repeated held-out resampling, preprocessing within each fold, calibration, and a high-specificity operating rule.

**Figure 1:**
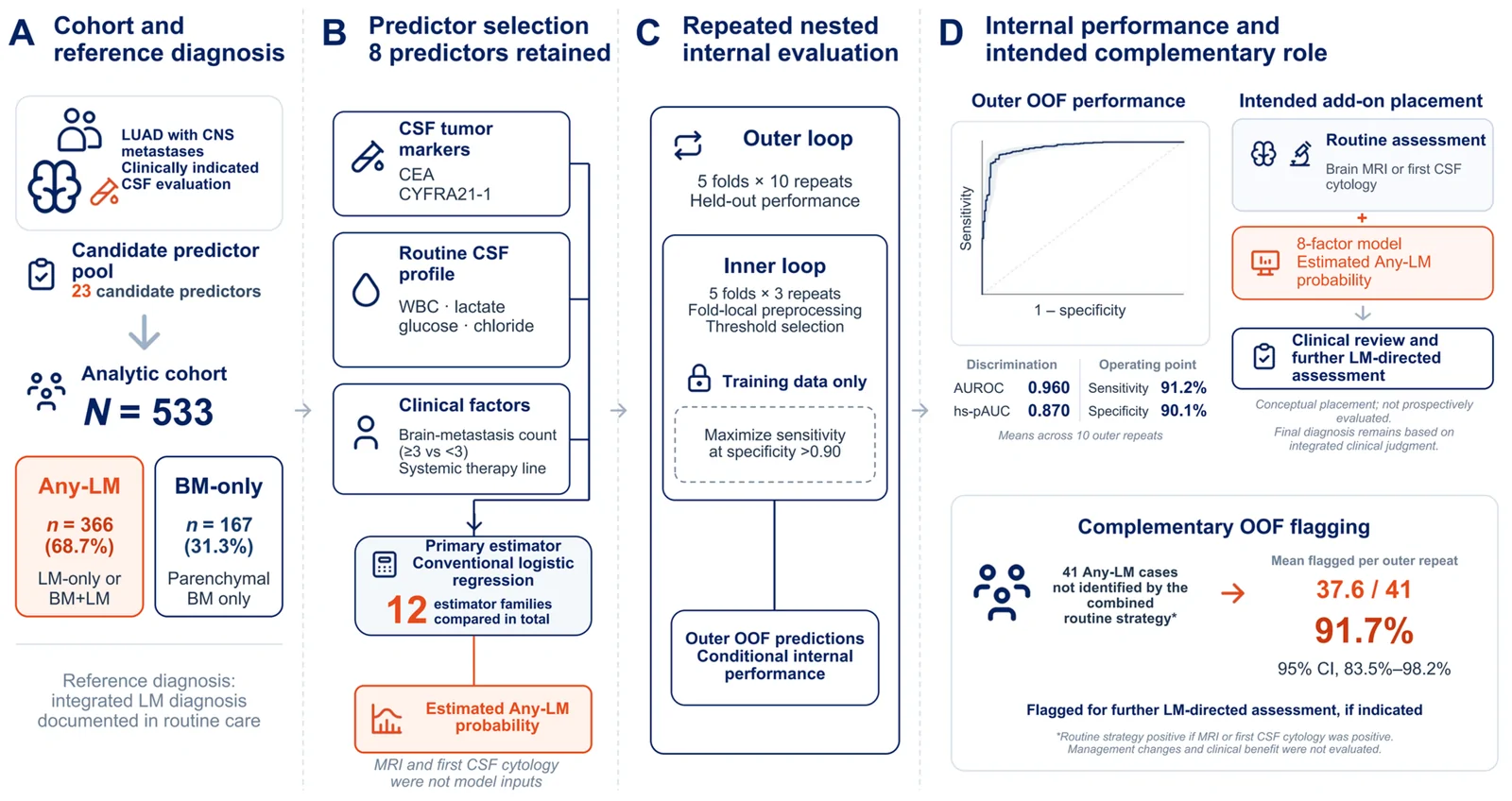
Study overview, final eight-factor model, repeated nested cross-validation, and intended complementary diagnostic role. The figure summarizes the selected clinical setting, final analytic cohort, eight clinical and CSF measurements available in the cohort, outer and inner resampling structure, held-out performance estimation, and illustrative diagnostic-pathway projection. Brain MRI and first CSF cytology were not model inputs. Complementary flagging among the 41 Any-LM patients not identified by either routine strategy was summarized across 10 repeated OOF classifications using split-specific inner-derived thresholds. The add-on pathway is conceptual and does not represent observed diagnostic confirmation or clinical benefit. Components may not sum to the total because of independent rounding. Reference basis for the 41 Any-LM cases: documented outside LM diagnosis before pretreatment CSF sampling at our center (*n* = 18) or confirmation by a later lumbar puncture (*n* = 23).

We hypothesized that continuous CSF variables measured in this cohort could capture a complementary multivariable pattern associated with current leptomeningeal involvement. Although no individual variable is specific to LM, joint variation in glucose, lactate, protein, cell counts, and tumor markers may reflect differences in metabolism, inflammation, and barrier function between BM-only and Any-LM. The target was contemporaneous current LM status, not subsequent LM development. We developed a conventional logistic model using the eight measurements retained during model development (CSF CEA, CYFRA21-1, WBC, lactate, glucose, chloride, brain metastasis burden, and systemic therapy line) to estimate the probability of a reference diagnosis of current leptomeningeal involvement (Any-LM) rather than BM-only. The final specification was internally evaluated using repeated nested cross-validation, with operating points derived exclusively from inner training predictions and performance estimated from outer held-out predictions. We examined calibration, sensitivity to alternative estimators and approaches to missing data, and descriptive complementarity relative to brain MRI and first CSF cytology within the reference diagnosis cohort. Secondary analyses included an illustrative diagnostic-pathway projection and exploratory analyses focused on lactate. We kept primary held-out estimates separate from the descriptive full cohort refit and exploratory extensions to distinguish their evidential roles. The model was intended to flag patients for further LM-directed diagnostic assessment, not to define LM, replace MRI or cytology, or establish clinical benefit. Figure 1 summarizes the study population, final eight-factor model, repeated nested internal evaluation, and intended complementary role.

## 2. Materials and Methods

### 2.1. Study Design and Setting

This single-center retrospective study included consecutive patients identified from routine clinical records at Beijing Tiantan Hospital, Capital Medical University, Beijing, China, between 1 January 2021 and 30 June 2026. The selected study population comprised patients with lung adenocarcinoma and central nervous system (CNS) metastatic involvement who underwent cerebrospinal fluid (CSF) evaluation during routine diagnostic care. The study developed and internally evaluated a multivariable model that distinguished a reference diagnosis of current leptomeningeal involvement (Any-LM) from brain metastasis without leptomeningeal involvement (BM-only). Reporting followed TRIPOD+AI, with diagnostic study items cross-checked against STARD [18,19]. The initial retrospective study was approved by the Medical Ethics Committee of Beijing Tiantan Hospital, Capital Medical University (KY2025-268-02; 19 November 2025). Separate ethics approval was subsequently granted under KY2026-193-02 (28 July 2026), whose approved scope includes the retrospective use of the existing clinical records analyzed in the present report from 1 January 2021 to 30 June 2026. The present report includes no prospectively collected data, and individual informed consent was waived for the retrospective analysis.

### 2.2. Participants, Routine Diagnostic Evaluation, and Cohort Assembly

The source population included patients undergoing clinically indicated CSF biochemical assessment during evaluation for suspected leptomeningeal metastasis (LM). Eligibility required a documented diagnosis of lung adenocarcinoma, qualifying CNS metastatic involvement, and sufficient clinical information to assign the case-level reference diagnosis. Among 790 screened records, 257 were excluded for one of three verified reasons: a diagnosis other than lung adenocarcinoma or an unconfirmed diagnosis of lung adenocarcinoma; absence of qualifying CNS metastatic involvement; or indeterminate LM status during the routine diagnostic evaluation. Counts for each exclusion reason were unavailable in the final analytic source and were therefore not assigned retrospectively. The final cohort contained 533 patients (Figure S1). The study size was determined by all eligible consecutive records available during the defined study period; statistical precision was quantified with 95% confidence intervals.

### 2.3. Reference Diagnosis and Retrospective Verification

The reference diagnosis contrasted Any-LM with BM-only. Any-LM included patients with current LM alone and those with concurrent parenchymal brain metastases and LM. The EANO–ESMO diagnostic framework informed interpretation of the documented clinical, radiographic, cytologic, and histopathologic evidence [4,5], but the study did not perform de novo blinded case-by-case classification or retrospectively assign a formal EANO–ESMO subtype to every patient. BM-only denoted parenchymal brain metastasis without documented leptomeningeal involvement.

In this retrospective study, LM status was derived primarily from the integrated diagnosis documented by treating clinicians during routine care. This diagnosis incorporated the clinical course, neurological findings, contrast-enhanced MRI, CSF cytology, including repeat sampling when performed, histopathology when available, and other longitudinal evidence. During data abstraction, one medical oncologist verified the recorded LM status; there was no second independent blinded review. Neither the model nor its threshold served as a formal diagnostic criterion. However, verification was not formally blinded to all candidate measurements or analysis outputs, so their influence cannot be excluded. We treated the classification as a retrospectively verified reference diagnosis with acknowledged uncertainty. Because MRI and CSF cytology contributed to the documented diagnostic process, comparisons with the model were descriptive and potentially subject to incorporation bias. Because some candidate CSF measurements may have been available to treating clinicians during routine diagnostic assessment, circularity between candidate predictors and the documented reference diagnosis cannot be excluded.

For strategy comparisons, first CSF cytology was defined as the result from the first lumbar puncture performed during the index LM diagnostic episode; repeat cytology could support the reference diagnosis but was not counted as the first-cytology strategy result. Among the 41 Any-LM patients not identified by either the brain MRI strategy or first CSF cytology, 18 had a documented diagnosis of LM at an outside institution before pretreatment CSF sampling at our center, whereas 23 had LM subsequently confirmed by a later lumbar puncture. These categories describe the clinical context of retrospective diagnostic verification; the model was developed to identify current LM status rather than predict future LM development. Among the 23 subsequently confirmed patients, the reference-diagnosis verification interval was defined from the date of the CSF sampling used for candidate-predictor measurement, at which first cytology did not identify LM, to the date of the first subsequent lumbar puncture that provided confirmatory evidence of LM.

### 2.4. Candidate Predictors and Routine Care Data Sources

The candidate pool contained 23 nonredundant clinical and CSF information units spanning demographics and disease course; CNS disease burden and prior treatment; CSF tumor markers; CSF cellular measures; and CSF biochemical measurements (Table S1). The pool included age; sex; coding of smoking history; driver gene category; line of systemic therapy; time since lung adenocarcinoma diagnosis; previous resection of the primary lung tumor; primary CNS metastasis; prior brain radiotherapy; number of brain metastases; CSF carcinoembryonic antigen (CEA), cytokeratin 19 fragment (CYFRA21-1), neuron-specific enolase, squamous cell carcinoma antigen, and progastrin-releasing peptide; CSF white blood cell (WBC) and total cell counts; one nonredundant polymorphonuclear/mononuclear composition measure; and CSF glucose, protein, lactate, chloride, and adenosine deaminase. Measurements came from retrospective clinical laboratory records at our center. Availability of specific assays, especially CSF tumor markers, may differ across institutions. Assay instruments, reagent manufacturers, and quality control procedures specific to each platform were not consistently retained in the analytic source and were not inferred.

The selected final model used eight variables: CSF CEA, CSF CYFRA21-1, CSF WBC count, CSF lactate, CSF glucose, CSF chloride, an indicator for at least three brain metastases, and line of systemic therapy. Brain MRI and first CSF cytology were not model inputs. For descriptive cohort summaries, unknown smoking status was grouped with no documented smoking history; other undocumented binary clinical fields followed the prespecified coding rules, and undocumented brain metastasis count was categorized as fewer than three. Full operational definitions and availability before imputation are provided in Table S1.

### 2.5. Missing Data and Preprocessing

The primary analysis used median imputation within each training partition on the original measurement scale. Within each resampling split, imputation parameters were estimated from the analysis partition and applied to its assessment partition. After imputation, CSF CEA, CYFRA21-1, WBC, and lactate were transformed as log2(x + 1). The six continuous CSF variables and line of systemic therapy were then standardized using the mean of the analysis partition and population standard deviation; the binary brain metastasis indicator was retained as 0 or 1 without standardization. Thus, data in the assessment partition were not used to estimate imputation, transformation, or scaling parameters. The observed missing data structure was summarized before imputation (Figure S2). Median imputation was retained as the primary preprocessing rule because it was deterministic, could be implemented within each training partition, preserved the full analytic cohort, and was compatible with subsequent application of the fitted model. It was not assumed to be statistically superior to multiple imputation.

As a formal missing-data sensitivity analysis, stochastic multiple imputation by chained equations was performed within every training partition using Bayesian-ridge conditional models with posterior sampling (20 imputations; 10 iterative rounds). CEA, CYFRA21-1, WBC, and lactate were imputed on the log2(x + 1) scale. The reference diagnosis was excluded from the imputation model, assessment observations did not estimate imputation parameters, and imputation, standardization, model fitting, and operating-point selection were repeated within the original nested cross-validation splits. For each held-out patient, predicted probabilities were averaged across the 20 imputations before performance assessment and threshold application.

Additional robustness analyses used two approaches (Figure S3). First, a single iterative imputation procedure using chained equations created one completed dataset per training partition (m = 1), using norm.predict for continuous variables, with a maximum of 10 iterations and a random seed of 20260710. The reference diagnosis was excluded from the imputation model, and rows in the assessment partition did not contribute to fitted imputation parameters. This procedure was a sensitivity analysis using single imputation and did not use Rubin pooling [20]. Second, a complete case analysis was performed in the 340 patients with all eight predictors observed. Because complete cases formed a smaller selected subset, this analysis was interpreted as supportive rather than directly comparable with the primary cohort.

### 2.6. Model Configurations and Final Model Specification

Model development began with 23 candidate predictors. Multiple candidate configurations were evaluated, including three specifications informed by stability (D1–D3), compact 10- and 9-factor configurations, the selected eight-factor configuration, and seven and six-factor configurations evaluated in sensitivity analyses (Table S2). These configurations were compared in terms of discrimination, calibration, parsimony, clinical availability, and predictor redundancy. The final eight-factor specification comprised CSF CEA, CYFRA21-1, WBC count, lactate, glucose, chloride, brain metastasis burden, and line of systemic therapy.

Using this fixed eight-factor specification, we compared 12 estimator families: conventional logistic regression, Firth/FLIC logistic regression, ridge, lasso, elastic net, logistic regression with splines, random forest, ExtraTrees, histogram gradient boosting, XGBoost, LightGBM, and support vector machine with a radial basis function kernel. Conventional logistic regression was selected as the primary estimator because its discrimination and calibration were comparable with those of the benchmark estimators, while its coefficients remained directly interpretable. Firth/FLIC and the other estimators served as benchmark or sensitivity analyses. Algorithm-specific hyperparameters were selected within the inner resampling loop when required; implementation and tuning details are provided in Table S2.

Predictor configuration development and selection of the primary estimator used information from the same cohort and were completed before the final repeated nested cross-validation runs, rather than repeated independently within every outer split. Therefore, the reported performance represents conditional internal validation and may retain optimism from configuration selection. Coefficients from the full cohort and the probability equation are reported descriptively in Table S3 and were not used to estimate held-out performance. Accordingly, repeated nested cross-validation does not constitute unbiased validation of a completely prespecified model, and independent external validation remains essential.

### 2.7. Repeated Nested Cross-Validation and Operating Point Selection

The final conventional logistic model underwent repeated nested stratified cross-validation, with model tuning confined to the inner resampling loop and performance assessment conducted in the outer held-out folds [21]. The outer loop used five folds repeated 10 times; within each outer analysis set, the inner loop used five folds repeated three times. The same predefined resampling splits were used consistently across estimators and sensitivity analyses. Each patient contributed one outer held-out probability in each repeat, yielding 5330 out-of-fold (OOF) records. Imputation, transformation, standardization, tuning, model fitting, and operating point derivation were confined to the relevant analysis partitions. Metrics for each repeat and operating thresholds for each split were retained to characterize resampling stability (Figure S4). The primary analysis used the verified reference diagnoses and repeated all preprocessing, model-fitting, threshold-selection, and cross-validation procedures.

For each of the 50 outer splits, a specificity-constrained operating point was derived from the corresponding OOF probabilities from the inner loop. Every unique OOF probability predicted in the inner loop was considered as a candidate threshold. Candidates were required to yield specificity strictly greater than 0.90; sensitivity was maximized among candidates satisfying this constraint, and the lowest threshold was selected when maximum sensitivity was tied. The selected threshold was then applied once to the associated outer held-out observations. If no candidate satisfied the specificity constraint, threshold selection would fail rather than invoke an empirical fallback; all 50 primary model splits satisfied the rule. The fixed threshold from the full cohort described below was never used to classify outer held-out predictions.

### 2.8. Performance Measures, Uncertainty Estimation, and Statistical Analyses

Performance was calculated within each of the 10 outer repeats using out-of-fold (OOF) predictions from held-out data and summarized as the mean across repeats. Discrimination was assessed using AUROC and standardized high-specificity partial AUROC over specificity 0.90–1.00 [22]. The Brier score, log loss, calibration intercept, and calibration slope were also evaluated [23,24]. Sensitivity, specificity, and balanced accuracy were calculated using thresholds derived exclusively from inner resampling for each outer split. Ninety-five percent confidence intervals were obtained using 5000 cluster bootstrap samples at the patient level, stratified by reference diagnosis group.

The ROC and decision curves were descriptive summaries of repeated OOF predictions, whereas the annotated estimates were derived from the primary analysis averaged across repeats. Decision curve analysis compared model-guided assessment with assessment of all or no patients [25]. It estimated model-based net benefit under assumed threshold preferences and did not measure changes in diagnostic behavior, treatment, or patient outcomes. A separate refit in the full cohort was used only to report model coefficients, adjusted odds ratios, the probability equation, a nomogram, and a fixed threshold, and did not contribute to held-out performance estimates.

Continuous variables were summarized as medians and interquartile ranges and compared using Mann–Whitney U tests. Categorical variables were summarized as counts and percentages and compared using Pearson chi-square or Fisher exact tests, as appropriate. These comparisons were descriptive and used available observations.

Exploratory lactate analyses evaluated the adjusted nonlinear association of lactate with Any-LM, the change in model performance after lactate removal, and partial correlations with selected CSF measurements within Any-LM. These analyses were hypothesis-generating and were not used to define a lactate cutoff or infer causality. Full statistical specifications are provided in the Supplementary Materials.

### 2.9. Comparison with Routine Diagnostic Strategies and Pathway Projection

Routine diagnostic strategies were defined from brain MRI and first CSF cytology recorded during the index diagnostic evaluation. First CSF cytology was the result from the first lumbar puncture performed during the index LM diagnostic episode; later cytology could support the reference diagnosis but was not counted in this strategy. The brain MRI strategy counted an unavailable or unperformed MRI as not identified in the pragmatic pathway; such patients were not labeled as false-negative MRI results. BM-only non-flagging rate was calculated as the proportion of all BM-only patients not flagged by the pragmatic strategy, including patients without an evaluable MRI examination. The combined routine strategy was positive when either brain MRI or first CSF cytology was positive. Strategy counts and diagnostic proportions were summarized within the reference diagnosis cohort (Table S4). Because both routine tests contributed to the documented integrated clinical diagnosis, these results were descriptive and were not interpreted as independent comparative accuracy estimates.

Complementary identification was evaluated from repeated OOF classifications among Any-LM patients not identified by either routine strategy. Separately, a descriptive refit in the full cohort was paired with a fixed threshold of 0.6103, derived from inner resampling during full model development, to illustrate a diagnostic-pathway projection. The scenario counted patients negative on both routine strategies who would be flagged for further LM-directed diagnostic assessment, potential additional Any-LM identifications, and BM-only patients flagged for further workup. The projection assumed that patients with a positive model result would undergo additional evaluation and that potential identification would require subsequent confirmation. It did not measure clinical behavior, diagnostic delay, treatment change, patient outcomes, or clinical benefit.

### 2.10. Software, Reproducibility, and Reporting Standards

All analyses were performed using Python 3.12.13 (Python Software Foundation, Beaverton, OR, USA) and R 4.6.0 (R Foundation for Statistical Computing, Vienna, Austria). Regression modeling and machine learning comparisons used scikit-learn, statsmodels, XGBoost, LightGBM, and logistf. Available package versions and implementation details are summarized in Table S2.

## 3. Results

### 3.1. Cohort Assembly and Analytic Framework

Of 790 screened records, 257 met one of the three exclusion categories, leaving 533 patients in the final analytic cohort (Figure S1). The reference diagnosis was Any-LM in 366 patients (68.7%), including LM alone and concurrent brain metastasis plus LM, and BM-only in 167 (31.3%). Thus, more than two-thirds of this cohort undergoing CSF evaluation had a reference diagnosis of current leptomeningeal involvement. Figure 1 summarizes the selected clinical setting, the final eight-factor model, the repeated nested cross-validation framework, and the model’s intended complementary role. The primary internal performance analysis generated 5330 OOF records from 10 outer repeats; descriptive analyses in the full cohort and exploratory analyses were kept separate.

### 3.2. Cohort Characteristics and Missing Data Patterns

Median age was 60.0 years (interquartile range [IQR], 53.0–66.0), and 316 of 533 patients (59.3%) were female (Table 1). Age, sex, coding of smoking history, and time since lung adenocarcinoma diagnosis were broadly similar between groups. Compared with BM-only, Any-LM was more often observed in patients receiving later lines of systemic therapy (median, 2 [IQR, 1–3] versus 1 [0–2]) and in those with at least three brain metastases (77.3% versus 53.9%). Prior brain radiotherapy and prior local treatment for brain metastases were less frequent in Any-LM than BM-only (36.6% versus 50.9% and 12.0% versus 26.3%, respectively).

**Figure 2:**
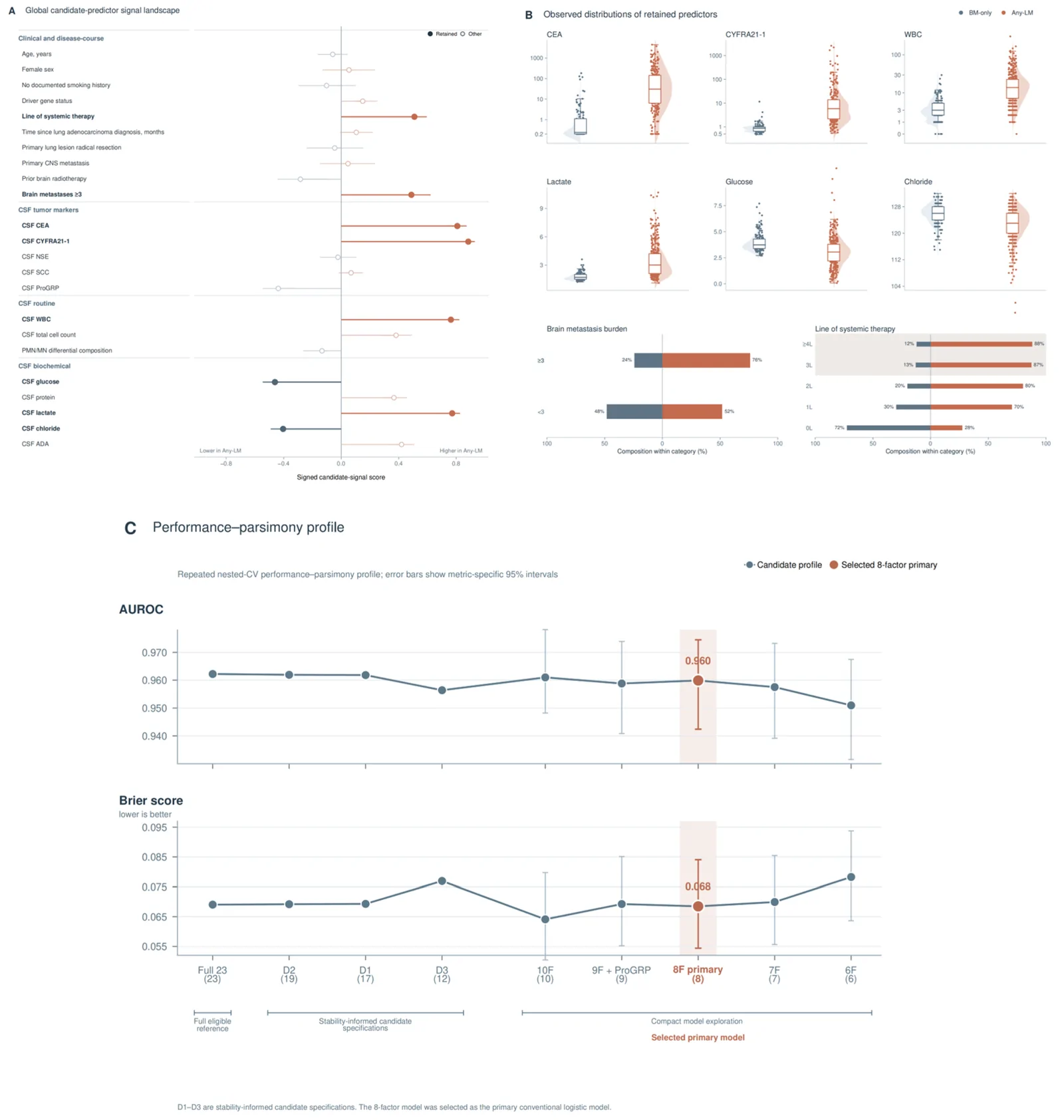
Candidate predictor signals and development of the eight-factor diagnostic model. (**A**) Signed univariable candidate signal scores across the eligible predictor pool; filled markers denote variables retained in the final model. (**B**) Distributions among available cases of retained continuous predictors and composition within categories of the two retained clinical predictors in Any-LM and BM-only. (**C**) Repeated cross-validated AUROC and Brier score profiles across the model development path. The final eight-factor specification was selected within this cohort after consideration of performance, robustness, interpretability, and predictor burden; the panel is not an independently validated model selection comparison. Error bars are 95% confidence intervals specific to each metric.

The retained continuous CSF measurements showed directionally consistent group separation (Figure 2, panels A and B). Median CSF CEA was 30.6 ng/mL (IQR, 6.32–143) in Any-LM and 0.24 ng/mL (0.20–1.10) in BM-only; corresponding values for CYFRA21-1 were 5.84 ng/mL (2.17–13.9) and 0.87 ng/mL (0.65–0.98). Any-LM also had higher WBC count (14.0 versus 3.0 cells/µL) and lactate (3.00 versus 1.70 mmol/L), and lower glucose (3.05 versus 3.74 mmol/L) and chloride (123 versus 126 mmol/L). All six comparisons had descriptive *p* values < 0.001. The distributions of the binary brain metastasis indicator and line of systemic therapy also differed between groups (Figure 2B).

CSF CEA and CYFRA21-1 were each missing in 169 patients (31.7%); missingness within each group was 32.5% in Any-LM and 29.9% in BM-only. CSF WBC was missing in 25 patients (4.7%), and lactate, glucose, and chloride were each missing in 23 (4.3%). The two clinical predictors had no missing values after application of the prespecified coding rules. The joint pattern of missingness and its distribution within each group are shown in Figure S2; these summaries did not establish a specific missing data mechanism. Missingness did not differ materially by reference diagnosis group but showed associations with some observed clinical factors. These descriptive analyses did not establish MCAR or MAR and could not exclude MNAR.

### 3.3. Selection and Specification of the Final Eight-Factor Model

The model development sequence began with 23 eligible information units, examined three candidate specifications informed by stability, and then evaluated compact 10-, 9-, 8-, 7-, and 6-factor configurations (Figure 2C and Table S2). The selected eight-factor specification retained CSF CEA, CYFRA21-1, WBC count, lactate, glucose, chloride, at least three brain metastases, and line of systemic therapy. It omitted CSF protein and ProGRP from the 10-factor extension and added the two clinical context variables to the six-factor CSF core. This specification was fitted with conventional unpenalized logistic regression; brain MRI and first CSF cytology were not model inputs. Because the configuration and primary estimator were selected using evidence from the same cohort before the final repeated resampling runs, subsequent OOF estimates represent conditional internal performance rather than evaluation of a configuration selected independently of the cohort.

### 3.4. Internal Cross-Validated Performance and Adjusted Predictor Associations

**Figure 3:**
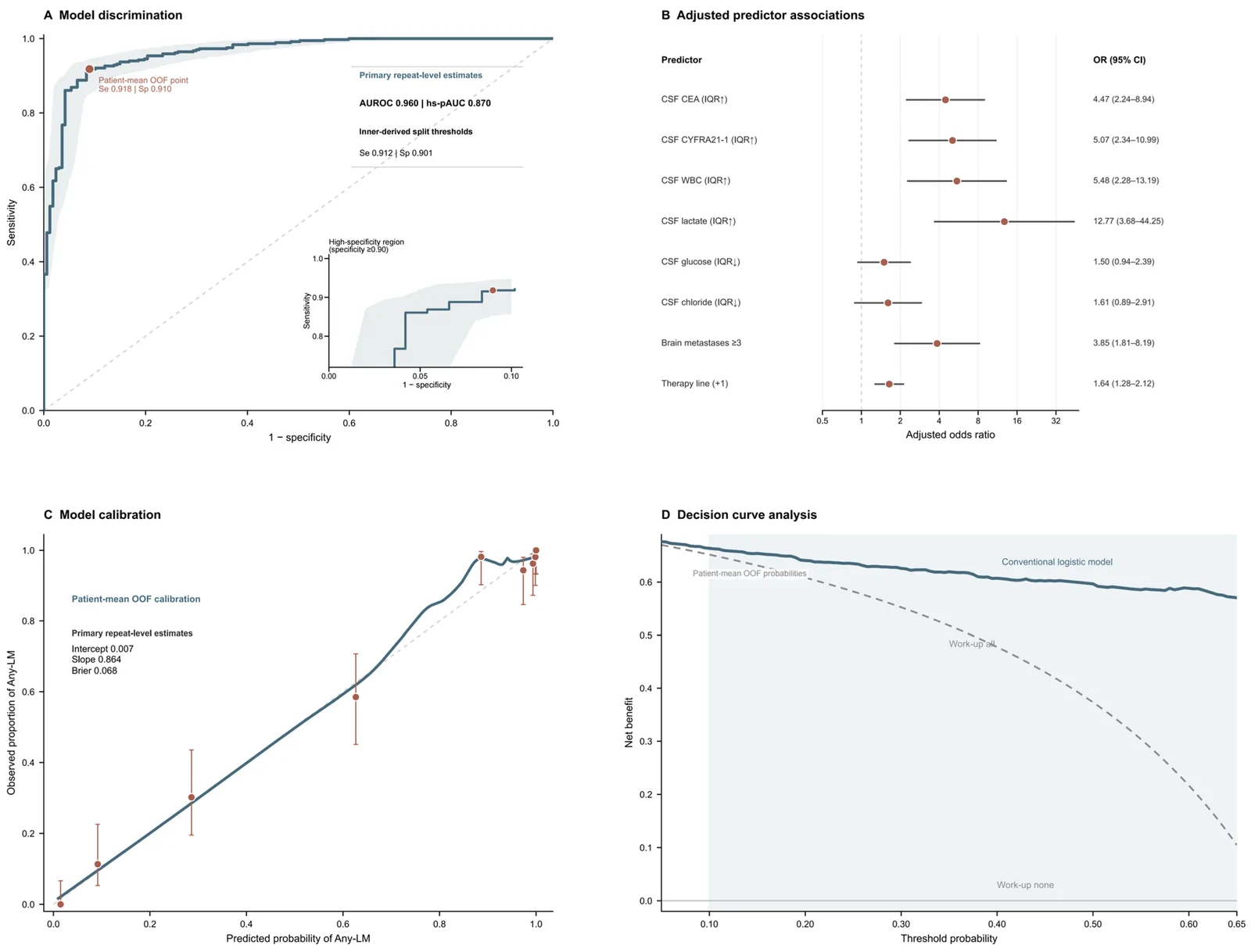
Conditional internal performance of the final eight-factor model and descriptive associations from the full cohort. (**A**) The ROC curve uses each patient’s mean of 10 outer held-out probabilities; the shaded band is the 95% bootstrap interval with clustering by patient. The red marker is a descriptive point selected on that same ROC based on each patient’s mean probability by maximizing sensitivity subject to specificity >0.90 (threshold, 0.6056; sensitivity, 0.918; specificity, 0.910). The blue text box reports the primary estimates averaged across repeats, including performance obtained with thresholds derived in the inner loop for each outer split; the graphical point is not the primary operating estimate. (**B**) Adjusted odds ratios from the descriptive refit in the full cohort, scaled to an increase (↑) or decrease (↓) across the interquartile range for continuous predictors. (**C**) Calibration points and the smooth curve use mean OOF probabilities for each patient, whereas the annotated intercept, slope, and Brier score are primary averages across repeats. (**D**) Decision curve analysis uses mean OOF probabilities for each patient. Panel (**B**) and the graphical point based on patient means are descriptive and are not outer held-out performance estimates averaged across repeats. Decision curve analysis estimates potential net benefit under the displayed threshold assumptions; it does not demonstrate improved diagnosis, management, or patient outcomes.

Across the 10 outer repeats, the final conventional logistic model had a mean AUROC of 0.960 (95% confidence interval [CI], 0.942–0.974) and standardized high-specificity partial AUROC of 0.870 (95% CI, 0.807–0.923) (Table 2 and Figure 3, panel A). Mean Brier score was 0.068 (95% CI, 0.054–0.084), and log loss was 0.238 (95% CI, 0.189–0.295). The calibration intercept was 0.007 (95% CI, −0.243 to 0.261), and the calibration slope was 0.864 (95% CI, 0.708–1.106). At the operating points for each split, sensitivity was 0.912 (95% CI, 0.883–0.938), specificity was 0.901 (95% CI, 0.857–0.939), and balanced accuracy was 0.907 (95% CI, 0.880–0.930). Panels A and C in Figure 3 display summaries based on each patient’s mean OOF probability; the numerical estimates above were calculated at the repeat level. The red marker in Figure 3A was derived separately on the displayed ROC based on each patient’s mean probability (threshold, 0.6056; sensitivity, 0.918; specificity, 0.910) and is a graphical summary rather than the primary operating estimate.

In the descriptive refit in the full cohort, higher transformed values of CEA, CYFRA21-1, WBC, and lactate, at least three brain metastases, and later therapy line were associated with greater odds of Any-LM (Figure 3, panel B). Adjusted odds ratios over an IQR increase were 4.47 (95% CI, 2.24–8.94) for CEA, 5.07 (2.34–10.99) for CYFRA21-1, 5.48 (2.28–13.19) for WBC, and 12.77 (3.68–44.25) for lactate. The odds ratio was 3.85 (1.81–8.19) for at least three versus fewer brain metastases and 1.64 (1.28–2.12) per additional therapy line. For decreases over their IQRs, the adjusted odds ratios were 1.50 (0.94–2.39) for glucose and 1.61 (0.89–2.91) for chloride. The OOF decision curve based on each patient’s mean probability estimated greater net benefit than assessment of none or all patients throughout the displayed threshold range (0.05–0.65) (Figure 3, panel D); this was a model-based utility estimate rather than evidence of improved patient outcomes.

### 3.5. Sensitivity and Robustness Analyses

The eight-factor model maintained high discrimination across alternative estimators, with AUROCs of 0.950–0.970 and high-specificity partial AUROCs of 0.853–0.901, although calibration varied more substantially (Table 2). Firth/FLIC logistic regression yielded results closely aligned with the primary conventional model, including an AUROC of 0.960, sensitivity of 0.912, and specificity of 0.901.

Formal multiple imputation performed separately within each training fold produced an AUROC of 0.957 (95% CI, 0.939–0.972), standardized high-specificity partial AUROC of 0.866 (0.808–0.920), Brier score of 0.073 (0.060–0.087), and calibration slope of 1.070 (0.879–1.375). At the operating point selected from inner OOF averaged probabilities, sensitivity was 0.892 (0.860–0.920) and specificity was 0.899 (0.855–0.940). Compared with primary median imputation, paired differences were −0.0030 (−0.0066 to 0.0006) for AUROC, +0.0048 (0.0010–0.0085) for Brier score, −0.020 (−0.033 to −0.008) for sensitivity, and −0.002 (−0.020 to 0.015) for specificity. Thus, discrimination and specificity were similar, whereas formal multiple imputation yielded a modestly higher Brier score and approximately two-percentage-point lower sensitivity. The chained-equations single-imputation and complete-case analyses yielded AUROCs of 0.967 and 0.969, respectively (Figure S3 and Table S5). Across the 10 primary cross-validation repeats, AUROC ranged from 0.9573 to 0.9619, and all 50 inner-loop selections met the prespecified specificity constraint.

### 3.6. Descriptive Complementarity with Brain MRI and First CSF Cytology

**Figure 4:**
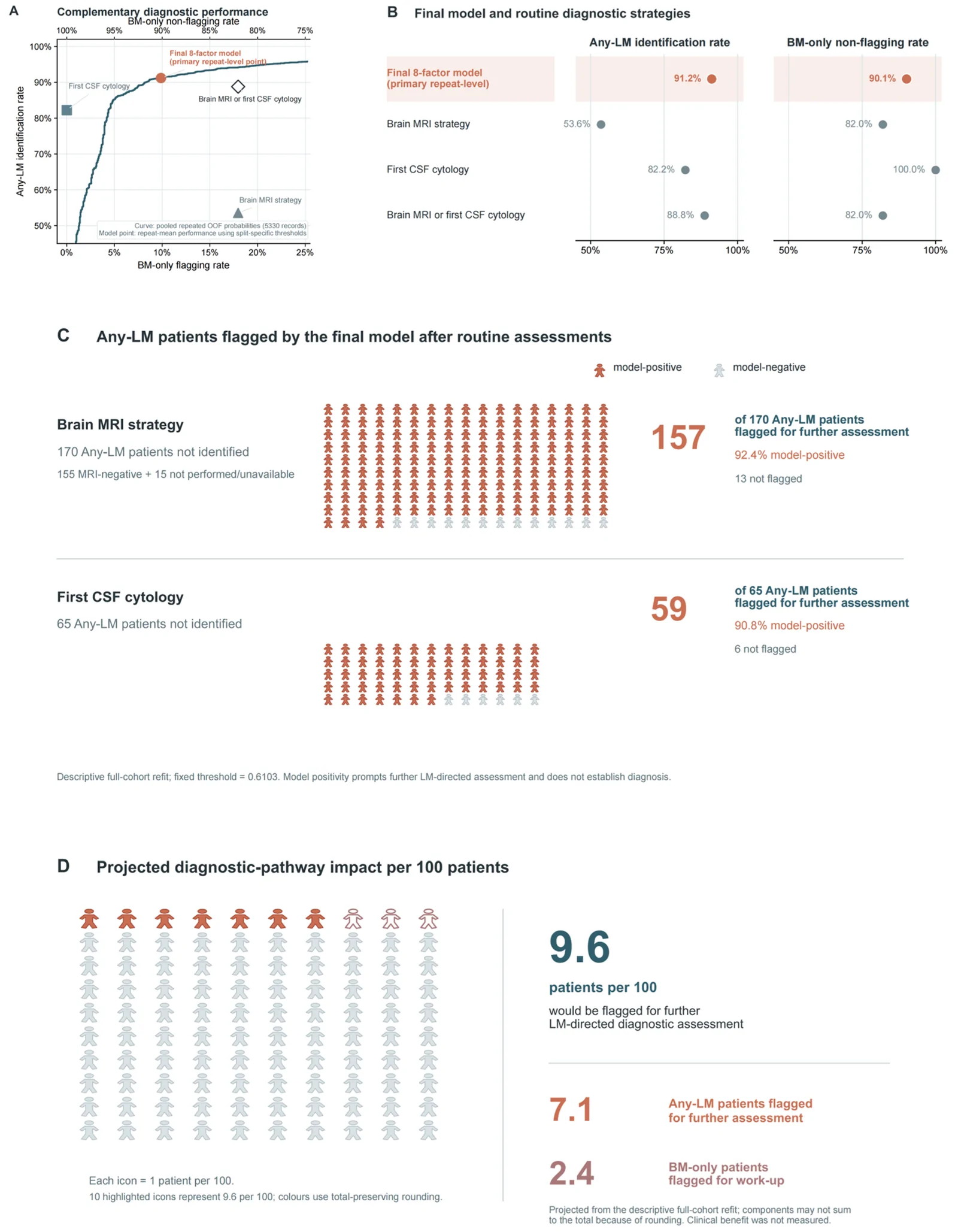
Descriptive positioning relative to routine diagnostic strategies and illustrative diagnostic-pathway projection. (**A**) The curve is a descriptive pooled ROC based on 5330 repeated outer held-out probabilities. The model point represents mean operating performance averaged across repeats using thresholds derived in the inner loop for each outer split and is not a coordinate based on a single threshold selected from the pooled curve; brain MRI, first CSF cytology, and their combination are shown using patient-level Any-LM identification rates and BM-only non-flagging rates. (**B**) Absolute Any-LM identification rates and BM-only non-flagging rates for the three routine strategies are shown alongside model sensitivity and specificity. These comparisons are descriptive, not independent head-to-head estimates of diagnostic accuracy, because MRI and cytology contributed to the reference diagnosis. MRI not performed or unavailable was counted as not identified by the pragmatic MRI strategy and not as an MRI test false-negative. (**C**) Among Any-LM patients not identified by the stated routine strategy, the descriptive refit in the full cohort at the fixed threshold of 0.6103 flagged 157/170 (92.4%) after the MRI strategy and 59/65 (90.8%) after first CSF cytology; the groups overlap. (**D**) Illustrative diagnostic-pathway counts per 100 patients from the descriptive refit in the full cohort. Model positivity indicates further evaluation for LM rather than diagnostic confirmation; the projection did not measure subsequent confirmation, clinical behavior, diagnostic delay, treatment change, patient outcomes, or clinical benefit. Separately rounded components may not sum to the total.

Within the reference diagnosis cohort, the pragmatic brain MRI strategy identified 196 of 366 Any-LM patients (identification rate, 53.6%) and did not flag 137 of 167 BM-only patients (strategy-level BM-only non-flagging rate, 82.0%). First CSF cytology identified 301 of 366 Any-LM patients (82.2%) and was negative in all 167 BM-only patients. The strategy based on brain MRI or first CSF cytology identified 325 of 366 Any-LM patients (88.8%) and did not flag 137 of 167 BM-only patients (BM-only non-flagging rate, 82.0%). The model based on repeated OOF predictions yielded sensitivity of 91.2% and specificity of 90.1% (Figure 4, panels A and B; Table S4). These values describe different strategies within the reference-diagnosis cohort and should not be interpreted as evidence that the model outperformed MRI or CSF cytology. Because the routine tests contributed to the documented integrated diagnosis, the comparisons are descriptive and subject to incorporation bias.

Forty-one unique Any-LM patients were not identified by either routine strategy. Across the 10 OOF repeats, the model flagged a mean of 37.6 of these patients for further evaluation for LM, corresponding to 91.7% (95% bootstrap CI with clustering by patient, 83.5–98.2%). In the separate descriptive refit in the full cohort, 157 of 170 Any-LM patients not identified by the pragmatic MRI strategy (92.4%) and 59 of 65 not identified by first CSF cytology (90.8%) were flagged for further assessment; these groups overlapped (Figure 4C). Of these 41 patients, 18 had a documented outside diagnosis of LM before pretreatment CSF sampling at our center, whereas 23 had LM confirmed by a subsequent lumbar puncture. Among the 23 patients whose reference LM diagnosis was confirmed by a subsequent lumbar puncture, the median interval from the CSF sampling used for candidate-predictor measurement to the confirmatory lumbar puncture was 23 days (IQR, 10.0–37.5 days; range, 5–52 days). Fifteen patients (65.2%) were confirmed within 30 days.

Using the full cohort threshold of 0.6103, 51 patients negative on both routine strategies were model positive: 38 had a reference diagnosis of Any-LM and 13 were BM-only. In an illustrative projection per 100 patients with the observed cohort prevalence, 9.6 would be flagged for further evaluation for LM, comprising 7.1 Any-LM and 2.4 BM-only patients (Figure 4D and Table S4); separately rounded components may not sum to the displayed total. These were scenario counts from the descriptive refit in the full cohort, not observed downstream diagnoses or outcomes, and any potential additional Any-LM identification would require subsequent diagnostic confirmation.

### 3.7. Exploratory Lactate Analyses

Among the eight retained predictors, CSF lactate showed the strongest adjusted association with Any-LM, motivating further exploratory evaluation of its diagnostic pattern and relationship with other CSF abnormalities. Lactate was available in 510 patients and was higher in Any-LM than in BM-only (median, 3.00 [IQR, 2.10–4.20] vs. 1.70 [IQR, 1.50–2.00] mmol/L; Figure S5A). After adjustment for the other seven predictors, lactate remained strongly associated with Any-LM (overall *p* = 4.90 × 10^−5^), with no evidence of departure from linearity (*p* = 0.337; Figure S5B). Across the observed IQR in the full cohort of 1.7–3.6 mmol/L, the adjusted odds ratio was 16.61 (95% CI, 3.61–76.42).

Removing lactate reduced overall AUROC by 0.0058 (eight-factor minus seven-factor difference; 95% CI, −0.0009 to 0.0134). The point estimate for high-specificity partial AUROC decreased by 0.0279 (95% CI, −0.0002 to 0.0615). Both confidence intervals included zero, and sensitivity and specificity at the selected operating point were essentially unchanged (Figure S5C). Within Any-LM, higher lactate was accompanied by higher WBC, protein, and CYFRA21-1 and by lower glucose and chloride (adjusted partial ρ, absolute range 0.446–0.550); its association with the cytologic proportion of atypical cells was inconclusive (Figure S5D and Table S6). These results are consistent with lactate marking a broader CSF biochemical disturbance associated with LM rather than acting as a standalone diagnostic marker. The analyses were exploratory and did not establish a mechanism or define a lactate cutoff.

## 4. Discussion

Brain parenchymal metastasis and leptomeningeal involvement represent anatomically and physiologically distinct patterns of CNS disease [1,3,4,26]. BM-only is centered within the brain parenchyma, whereas LM involves the leptomeninges and CSF pathways. This anatomical distinction permits more direct interaction among malignant cells, host responses, and the CSF compartment [1,3,4]. In our cohort, Any-LM differed from BM-only across tumor markers, cellular measures, metabolic indices, and homeostatic measurements. Similar abnormalities have been reported in clinical CSF and metabolomic studies [9,10,11,17]. These group differences provide the quantitative basis for the model, but they do not establish a distinct biological entity.

The model summarizes a multivariable clinical and CSF measurement pattern rather than a new definition of LM. It integrates tumor marker signals (CEA and CYFRA21-1), cellular and metabolic disturbance (WBC and lactate), altered CSF homeostasis (glucose and chloride), and CNS disease and treatment context (brain metastasis burden and line of systemic therapy). Each component is incomplete and nonspecific when considered alone. Together, they retain quantitative information that would be lost if LM-associated abnormalities were reduced to separate laboratory cutoffs or to the presence or absence of radiographic enhancement or malignant cells. The estimated probability quantifies concordance with this multidomain pattern. It does not directly measure tumor burden and cannot substitute for the categorical clinical diagnosis. It is a contemporaneous classification model for current LM involvement in patients already undergoing CSF evaluation, not a model of future LM onset or earlier preclinical prediction.

Lactate illustrates both the scope and the limitations of this measurement pattern. It showed the strongest adjusted association with Any-LM among the eight retained predictors. Within Any-LM, higher lactate was accompanied by higher WBC, protein, and CYFRA21-1 and by lower glucose and chloride, indicating a broader disturbance of the sampled CSF compartment rather than an isolated biomarker abnormality. The observed constellation is compatible with changes in tumor and immune cell metabolism, barrier function, and CSF flow. Independent metabolomic studies have also identified structured biochemical differences in CSF from patients with LM [17,27,28]. However, these mechanisms were not measured directly, and infection, nonmalignant inflammation, treatment exposure, sampling site, and preanalytic handling can produce overlapping changes [11,29]. Removing lactate caused only modest performance changes whose confidence intervals included zero. Lactate should be considered one component of the correlated CNS–CSF measurement pattern, not a causal driver, an independently confirmatory marker, or a basis for a standalone threshold.

The multidomain pattern also explains why the model may complement MRI and first CSF cytology. MRI depicts anatomical abnormalities, cytology samples malignant cell shedding at a particular lumbar puncture, and CSF cellular and biochemical measurements characterize sampled-compartment disturbance. Because these manifestations may be spatially heterogeneous and need not appear synchronously, a negative MRI or first cytology result does not exclude LM when clinical suspicion persists [4,5,7,8]. In this cohort, 41 unique Any-LM cases were not identified by the combined routine strategy, and the repeated held-out model flagged a mean of 37.6 (91.7%) for further evaluation for LM. This finding indicates complementarity, not diagnostic superiority. MRI and cytology contributed to the integrated clinical reference diagnosis, making the strategy comparisons susceptible to incorporation bias. A high model probability could prompt consideration of imaging review, repeat or optimized cytology, spinal imaging when indicated, or a specialized CSF assay. It should not confirm LM or replace integrated clinical judgment.

This study differs from earlier models and specialized CSF assays in both population and intended use. Previous diagnostic models included heterogeneous solid tumors, emphasized clinicopathologic risk factors in lung adenocarcinoma, or evaluated an NSCLC tumor marker panel [12,13,14]. We focused on current Any-LM rather than BM-only among patients with lung adenocarcinoma, established CNS metastatic involvement, and a clinical indication for CSF evaluation. The model combined tumor markers with cellular, biochemical, and clinical context measurements, and repeated held-out evaluation characterized its variability without relying on a single random split. By contrast, CSF circulating tumor cells, cell-free DNA, and metabolomics can provide more specific disease detection and molecular characterization [16,17,30,31]. These methods require specialized platforms, assay-specific interpretation, and additional resources. These approaches are complementary: the present model, based on clinical variables and available CSF measurements, may help prioritize further assessment, while advanced CSF assays can provide additional cellular, genomic, or metabolic evidence when available.

**Figure 5:**
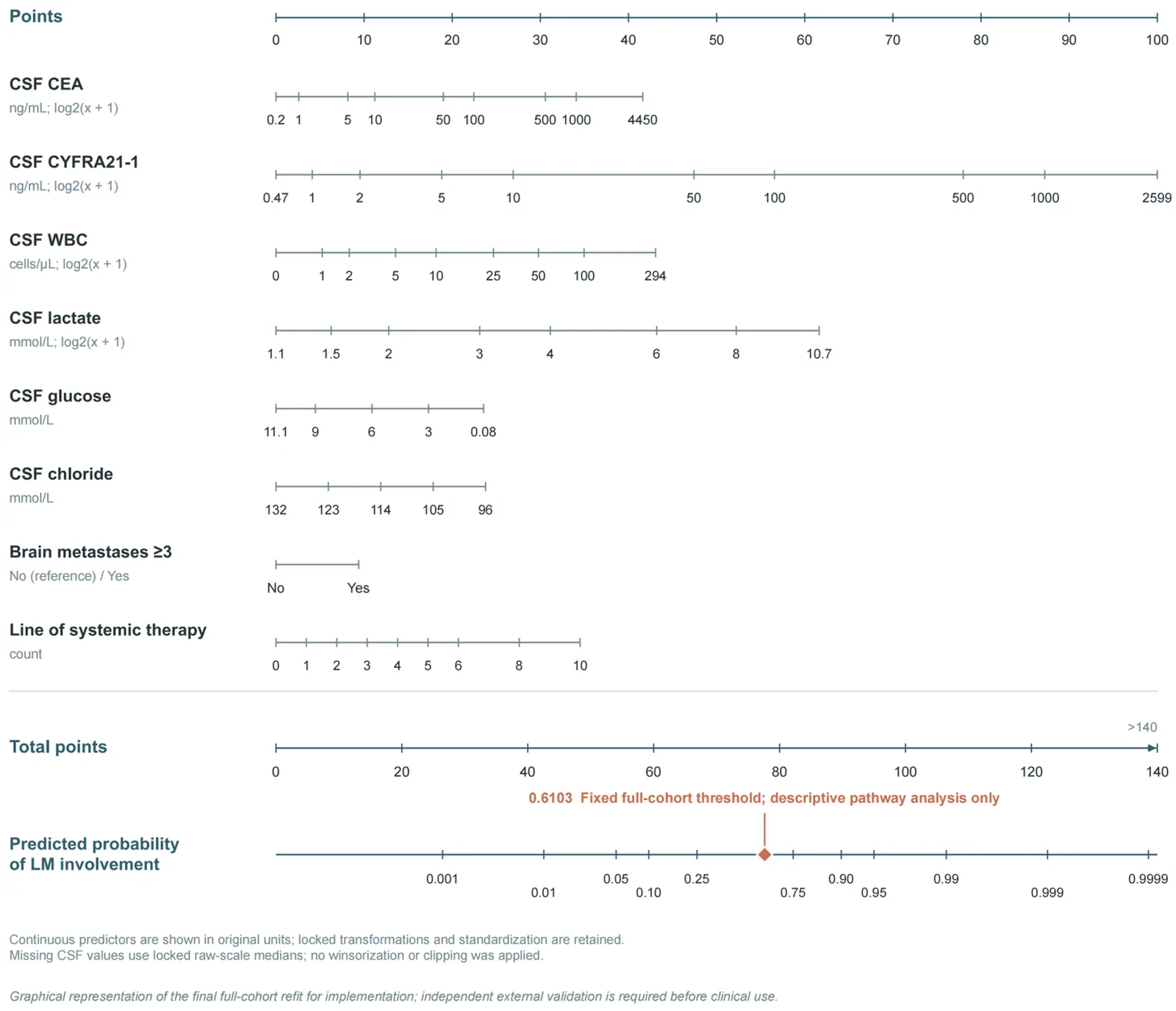
Nomogram derived from the descriptive full-cohort refit of the final eight-factor logistic model. This refit illustrates model implementation and was not used to estimate outer held-out performance. The nomogram is not an externally validated clinical decision tool and requires independent validation before clinical use.

The intended clinical role informed the operating rule. We favored sensitivity while requiring specificity greater than 0.90 in the inner OOF predictions because a positive model result would prompt further assessment, not immediate treatment or diagnostic confirmation. Repeated outer held-out estimates showed strong discrimination and approximately 90% sensitivity and specificity, and decision curve analysis estimated potential net benefit under the displayed threshold assumptions; it did not demonstrate improved diagnosis, management, or patient outcomes. However, nested resampling remains an internal evaluation and does not establish transportability to an independent setting [21,32]. Predictor and estimator choices were informed by the same cohort, the calibration slope below one suggests some overfitting, and split-specific thresholds describe an internal evaluation procedure rather than a portable clinical cutoff [24,25]. The fixed full cohort threshold and projected pathway counts are also descriptive. Their predictive values and consequences depend on prevalence, referral patterns, assay calibration, and downstream practice. The model should be regarded as an investigational triage layer within the established LM diagnostic pathway. Similar estimates across several algorithms do not eliminate residual optimism from cohort-informed predictor and model specification. For transparent calculation, Figure 5 presents a nomogram from the descriptive full-cohort refit; it illustrates model implementation and is not an externally validated clinical decision tool.

Several limitations materially constrain interpretation. This was a highly selected single-center cohort from a Chinese tertiary referral center and included only patients with lung adenocarcinoma, established CNS metastatic involvement, and clinically indicated lumbar puncture; 68.7% had Any-LM and 81.1% had an EGFR alteration. Performance, predictive values, and assay relationships may therefore differ in populations with other histologies, molecular epidemiology, referral pathways, LM prevalence, or laboratory platforms. LM status was derived primarily from the documented integrated clinical diagnosis and verified retrospectively by one medical oncologist rather than an independent blinded panel. MRI and cytology contributed to the reference diagnosis, creating incorporation bias, and candidate CSF measurements may also have influenced routine diagnostic verification, creating possible circularity. The retained measurements arose during the same clinical episode, so the analysis addresses current LM status and cannot establish that every predictor preceded every diagnostic decision. Repeat lumbar puncture was clinically driven rather than protocol-mandated. Therefore, the 23 subsequently confirmed patients constituted a selectively verified subgroup with relatively high clinical suspicion, whereas patients with milder or unrecognized LM who were not retested could not be captured. The observed interval should not be interpreted as the real-world duration of diagnostic delay or as time potentially saved by the model. Delayed confirmation may also have introduced temporal misclassification into the 91.7% complementary flagging estimate, which is conditional on the retrospectively verified reference diagnosis. CEA and CYFRA21-1 were missing in 31.7% of patients; their availability varies across centers, and these assays are not part of routine CSF panels in some settings. The data could not establish MCAR or MAR or exclude MNAR; formal multiple imputation assumed conditional MAR and assessed procedural robustness rather than proving a noninformative missingness mechanism. Median imputation may understate imputation uncertainty. Cohort-informed model specification may have left residual optimism despite repeated nested cross-validation. Finally, the illustrative pathway projection and decision curve did not measure diagnostic confirmation, delays, additional procedures, management changes, harms, quality of life, survival, or clinical benefit.

Further evaluation should proceed in stages. A multicenter external study should enroll consecutive patients at the point of CSF evaluation, retain indeterminate cases, prespecify predictor timing and assay handling, and include centers with different referral patterns and LM prevalences. It should examine calibration-in-the-large, calibration slope, discrimination, high-specificity operating performance, and net benefit with adequate precision [32]. Recalibration or model updating should be separated from evaluation of the original model. If transportability is acceptable, a prospective impact study should then test whether model-guided assessment changes diagnostic actions, time to diagnosis, unnecessary workup, patient burden, or outcomes relative to usual care [33]. Overall, the retrospectively verified reference diagnosis was associated with a graded pattern across the evaluated clinical and CSF measurements. This pattern may complement MRI, cytology, and integrated clinical judgment but cannot replace them.

## 5. Conclusions

In this selected cohort of patients with lung adenocarcinoma, CNS metastatic involvement, and clinically indicated CSF evaluation, the evaluated clinical and CSF measurements captured a multivariable pattern associated with the retrospectively verified reference diagnosis of current LM involvement. The eight-factor model showed strong conditional internal performance and may provide an investigational adjunct for further LM-directed assessment. It does not confirm LM or replace MRI, cytology, and integrated clinical judgment. Independent multicenter validation and prospective impact evaluation are required before clinical implementation.

## Figures and Tables

**Table 1:** Clinical and cerebrospinal fluid characteristics of the reference diagnosis cohort by diagnostic group.

Characteristic	Overall (*n* = 533)	Any-LM (*n* = 366)	BM-only (*n* = 167)	*p* Value
**Clinical characteristics**				
Age, years	60.0 (53.0–66.0)	59.5 (53.0–66.0)	60.0 (54.0–66.0)	0.275
Female sex	316/533 (59.3)	220/366 (60.1)	96/167 (57.5)	0.567
Never smoker/no documented smoking history	377/533 (70.7)	254/366 (69.4)	123/167 (73.7)	0.317
**Driver gene status**				0.018
EGFR exon 19 deletion	157/533 (29.5)	105/366 (28.7)	52/167 (31.1)	
EGFR L858R	225/533 (42.2)	165/366 (45.1)	60/167 (35.9)	
Uncommon EGFR alteration	50/533 (9.4)	39/366 (10.7)	11/167 (6.6)	
Other driver alteration	63/533 (11.8)	37/366 (10.1)	26/167 (15.6)	
No known driver alteration/not tested	38/533 (7.1)	20/366 (5.5)	18/167 (10.8)	
Line of systemic therapy	2 (1–3)	2 (1–3)	1 (0–2)	<0.001
Time since LUAD diagnosis, months	30.0 (14.0–52.0)	32.0 (17.1–52.0)	25.6 (10.0–51.2)	0.052
**CNS disease burden and prior treatment**				
Brain metastases ≥ 3	373/533 (70.0)	283/366 (77.3)	90/167 (53.9)	<0.001
Prior brain radiotherapy	219/533 (41.1)	134/366 (36.6)	85/167 (50.9)	0.002
Prior local treatment for brain metastases	88/533 (16.5)	44/366 (12.0)	44/167 (26.3)	<0.001
Primary lung lesion radical resection	153/533 (28.7)	103/366 (28.1)	50/167 (29.9)	0.670
Primary CNS metastasis	177/533 (33.2)	124/366 (33.9)	53/167 (31.7)	0.626
**CSF tumor markers**				
CSF CEA, ng/mL	9.16 (0.53–70.4)	30.6 (6.32–143)	0.24 (0.20–1.10)	<0.001
CSF CYFRA21-1, ng/mL	2.38 (0.94–8.71)	5.84 (2.17–13.9)	0.87 (0.65–0.98)	<0.001
CSF NSE, ng/mL	16.3 (11.8–22.8)	16.2 (11.4–23.1)	17.1 (12.4–22.4)	0.727
CSF SCC, ng/mL	0.10 (0.10–0.10)	0.10 (0.10–0.10)	0.10 (0.10–0.10)	0.131
CSF ProGRP, pg/mL	191 (135–253)	176 (117–221)	235 (188–314)	<0.001
**CSF routine examination**				
CSF white blood cell count, cells/µL	8.00 (3.00–18.0)	14.0 (7.00–23.0)	3.00 (2.00–5.00)	<0.001
CSF total cell count, cells/µL	80.0 (6.00–158)	104 (11.0–208)	5.00 (2.00–104)	<0.001
CSF polymorphonuclear cells, %	9.50 (0.00–28.6)	8.30 (0.00–18.6)	25.0 (0.00–50.0)	0.019
CSF mononuclear cells, %	90.5 (71.4–100)	91.7 (81.5–100)	75.0 (50.0–100)	0.019
**CSF biochemical profile**				
CSF glucose, mmol/L	3.36 (2.59–4.05)	3.05 (2.20–3.80)	3.74 (3.39–4.30)	<0.001
CSF protein, mg/dL	49.8 (33.8–79.6)	58.8 (37.8–99.9)	38.3 (30.9–52.2)	<0.001
CSF lactate, mmol/L	2.30 (1.70–3.60)	3.00 (2.10–4.20)	1.70 (1.50–2.00)	<0.001
CSF chloride, mmol/L	124 (122–127)	123 (120–126)	126 (124–128)	<0.001
CSF adenosine deaminase, U/L	0.50 (0.30–0.90)	0.60 (0.30–1.00)	0.30 (0.20–0.50)	<0.001

Notes: Data are median (IQR) or *n*/*N* (%). Continuous variables were compared using two-sided Mann–Whitney U tests; categorical variables were compared using Pearson’s chi-square or Fisher’s exact tests, as appropriate. *p* values are descriptive and were not adjusted for multiplicity. Continuous summaries use available observations. Any-LM includes LM alone and concurrent brain metastasis plus LM. BM-only denotes brain metastasis without LM.

**Table 2:** Repeated nested cross-validated performance of the final eight-factor clinical and CSF model across estimators.

Model	AUROC (95% CI)	hs-pAUC (95% CI)	Brier Score (95% CI)	Calibration Slope (95% CI)	Sensitivity at the Specificity Constrained Threshold Derived in the Inner Loop (95% CI)	Outer Held-Out Specificity at the Corresponding Threshold (95% CI)
**Primary model**						
**Conventional logistic regression**	**0.960 (0.942–0.974)**	**0.870 (0.807–0.923)**	**0.068 (0.054–0.084)**	**0.864 (0.708–1.106)**	**0.912 (0.883–0.938)**	**0.901 (0.857–0.939)**
**Logistic regression sensitivity/reference models**						
Firth/FLIC logistic regression	0.960 (0.942–0.974)	0.870 (0.806–0.923)	0.069 (0.055–0.084)	0.926 (0.760–1.184)	0.912 (0.883–0.938)	0.901 (0.857–0.938)
Ridge logistic regression	0.950 (0.931–0.966)	0.853 (0.784–0.912)	0.094 (0.085–0.104)	1.882 (1.495–2.493)	0.917 (0.889–0.942)	0.905 (0.862–0.943)
Lasso logistic regression	0.959 (0.941–0.974)	0.868 (0.805–0.923)	0.069 (0.056–0.084)	0.989 (0.811–1.264)	0.914 (0.886–0.939)	0.899 (0.856–0.937)
Elastic net logistic regression	0.957 (0.939–0.972)	0.863 (0.798–0.920)	0.077 (0.066–0.090)	1.465 (1.182–1.916)	0.918 (0.890–0.943)	0.898 (0.854–0.938)
**Nonlinear logistic reference**						
RCS/GAM logistic regression	0.957 (0.939–0.972)	0.863 (0.798–0.918)	0.071 (0.057–0.087)	0.879 (0.729–1.103)	0.904 (0.874–0.930)	0.898 (0.854–0.936)
**Machine learning benchmarks**						
Random Forest	0.970 (0.954–0.982)	0.900 (0.843–0.943)	0.063 (0.051–0.075)	1.267 (0.950–1.735)	0.919 (0.893–0.943)	0.896 (0.850–0.935)
ExtraTrees	0.968 (0.953–0.980)	0.901 (0.849–0.938)	0.089 (0.082–0.098)	3.013 (2.562–3.671)	0.913 (0.883–0.939)	0.904 (0.859–0.942)
HistGradientBoosting	0.968 (0.951–0.981)	0.886 (0.819–0.937)	0.060 (0.047–0.075)	0.939 (0.810–1.124)	0.919 (0.893–0.942)	0.900 (0.856–0.937)
XGBoost	0.969 (0.953–0.982)	0.896 (0.830–0.943)	0.061 (0.049–0.075)	1.119 (0.954–1.371)	0.915 (0.887–0.940)	0.914 (0.874–0.949)
LightGBM	0.970 (0.955–0.982)	0.894 (0.834–0.940)	0.059 (0.047–0.074)	0.996 (0.861–1.202)	0.924 (0.898–0.946)	0.900 (0.856–0.938)
RBF-SVM	0.961 (0.941–0.977)	0.860 (0.773–0.928)	0.065 (0.051–0.080)	0.985 (0.804–1.234)	0.920 (0.892–0.945)	0.896 (0.852–0.935)

Notes: All 12 estimators used the same eight-predictor set. Point estimates are arithmetic means of metrics calculated separately in each of the 10 outer repeats. The 95% confidence intervals were obtained from 5000 patient cluster bootstrap resamples stratified by reference diagnosis group, retaining all 10 repeated predictions and classifications for each sampled patient. The specificity constraint was applied to inner OOF predictions within each outer training set; outer held-out specificity at the corresponding threshold was not required to remain strictly above 0.90. The conventional unpenalized logistic regression model was primary; the other estimators were benchmark or sensitivity analyses. hs-pAUC denotes standardized partial AUROC over specificity 0.90–1.00; Sp, specificity.

## Data Availability

Individual-level records, OOF probabilities, and fold assignments are not publicly available because of privacy and ethics restrictions. The analysis code is not publicly available. The complete model equation and preprocessing constants are provided in Table S3, and the resampling, threshold-selection, and uncertainty procedures are described in Section 2 and Supplementary Materials.

## Supplementary Materials

The following supporting information can be downloaded at: https://www.mdpi.com/article/10.3390/diagnostics16172742/s1, Supplementary Methods S1: Operational definitions and data-coding rules; Supplementary Methods S2: Candidate-configuration development and specification of the final model; Supplementary Methods S3: Repeated nested cross-validation and statistical implementation; Supplementary Methods S4: Sensitivity and exploratory analyses; Figure S1: Study population flow; Figure S2: Missing-data structure among the final eight predictors; Figure S3: Sensitivity of model performance to missing-data handling; Figure S4: Stability of repeated nested cross-validation; Figure S5: Exploratory lactate-focused analyses; Table S1: Definitions, availability, and preprocessing of all candidate predictors; Table S2: Predictor-set development and estimator benchmark; Table S3: Final full-cohort model coefficients and probability equation; Table S4: Strategy-level performance, complementary flagging, and illustrative pathway projection; Table S5: Sensitivity analyses; Table S6: Lactate-centered partial-rank-correlation results in observed Any-LM pairs.

## Abbreviations

The following abbreviations are used in this manuscript:

Any-LM	Current leptomeningeal involvement, including LM alone and concurrent brain metastasis plus LM
AUROC	Area under the receiver operating characteristic curve
BM	Brain metastasis
BM-only	Brain metastasis without leptomeningeal metastasis
CEA	Carcinoembryonic antigen
CI	Confidence interval
CNS	Central nervous system
CSF	Cerebrospinal fluid
CTC	Circulating tumor cell
CYFRA21-1	Cytokeratin 19 fragment
FLIC	Firth logistic regression with intercept correction
hs-pAUC	Standardized high-specificity partial area under the receiver operating characteristic curve
IQR	Interquartile range
LM	Leptomeningeal metastasis
LUAD	Lung adenocarcinoma
MRI	Magnetic resonance imaging
OOF	Out-of-fold
RCS	Restricted cubic spline
WBC	White blood cell
