# Supplementary material for: Clinical and CSF Measurements for Identification of Leptomeningeal Metastasis in Lung Adenocarcinoma
Source: Diagnostics (Basel). 2026 Aug 27;16(17):2742. doi: 10.3390/diagnostics16172742 (PMC13565679; doi:10.3390/diagnostics16172742)
Supplement: Supplementary file 1 [file diagnostics-16-02742-s001.zip › diagnostics-4487830-supplementary.pdf]

Clinical and CSF Measurements for Identification of Leptomeningeal Metastasis in Lung Adenocarcinoma

Supplementary Methods S1–S4 | Figures S1–S5 | Tables S1–S6

Supplementary Methods

S1. Operational definitions and data-coding rules

The routine diagnostic evaluation was the clinical episode during which the retained cerebrospinal fluid (CSF) measurements and diagnostic evidence were recorded. The analytic dataset contained one record per patient. Because a common numeric sampling window and complete records of how repeated measurements were selected were unavailable across data domains, no retrospective window was imposed. The retained value was analyzed without post hoc averaging or selection, and an absent field was treated as missing. The exact temporal order of all measurements and diagnostic decisions could not be verified. Accordingly, the analyses classify contemporaneous current LM status and cannot establish that every biomarker measurement preceded every routine diagnostic decision.

The reference diagnosis contrasted Any-LM with BM-only. Any-LM included LM alone and concurrent brain metastasis (BM) plus LM; BM-only denoted parenchymal BM without documented LM. The EANO–ESMO framework informed interpretation of the documented clinical, radiographic, cytologic, and histopathologic evidence. However, the study did not perform de novo blinded case-by-case classification or retrospectively assign a formal EANO–ESMO subtype to every patient. LM status was based primarily on the integrated clinical diagnosis documented during routine care and was verified by one medical oncologist during data abstraction, without a second independent blinded review. Neither the model nor its threshold was a formal diagnostic criterion. Because verification was not formally blinded to all candidate measurements or analysis outputs, their influence cannot be excluded. The classification was treated as a retrospectively verified reference diagnosis with acknowledged uncertainty.

For strategy comparisons, first CSF cytology was defined as the result from the first lumbar puncture performed during the index LM diagnostic episode; repeat cytology could support the reference diagnosis but was not counted as the first-cytology strategy result. Among the 41 Any-LM patients not identified by either the brain MRI strategy or first CSF cytology, 18 had a documented diagnosis of LM at an outside institution before pretreatment CSF sampling at our center, whereas 23 had LM subsequently confirmed by a later lumbar puncture. These categories describe the context of retrospective verification rather than prospective prediction.

Brain MRI was classified as positive, negative, or unavailable/not performed. For the pragmatic MRI strategy, unavailable examinations were counted as not identified, but not as negative tests or MRI false negatives. The combined routine strategy was positive when either MRI or first CSF cytology was positive. Because both tests informed the documented integrated clinical diagnosis, comparisons were descriptive strategy analyses within the reference diagnosis cohort.

Brain metastasis burden was coded 1 for at least three metastases and 0 for fewer than three or an undocumented count. According to the prespecified coding rules, an unrecorded systemic therapy line was coded as 0; these variables were complete only after applying these rules. Laboratory values were analyzed in their recorded units, and availability was assessed before imputation. Assay platforms, reagents, detection limits, reference intervals, and platform-specific quality control metadata were not consistently retained and were not inferred.

S2. Candidate-configuration development and specification of the final model

The development pool contained the 23 nonredundant information units in Table S1. Developmental stability sets D1–D3 were generated within the original development splits using predefined rules for selection frequency and sign consistency. These sets informed, but did not define, the final model. The compact development path compared 10-, 9-, 8-, 7-, and 6-factor configurations. The selected eight-factor specification included CSF CEA, CYFRA21-1, WBC, lactate, glucose, chloride, at least three brain metastases, and systemic therapy line (Table S2A).

Performance, calibration, parsimony, clinical availability, and redundancy informed the final specification. These decisions used the same 533-patient cohort and preceded the final repeated nested cross-validation runs. Selection of the predictor configuration and primary estimator was not repeated within each outer split. The outer held-out estimates represent conditional internal cross-validated performance and may retain selection optimism. Thus, the repeated nested estimates are conditional internal performance after cohort-informed development and are not unbiased validation of a completely prespecified model.

Twelve estimator families were benchmarked using the same eight predictors (Table S2B). Conventional unpenalized logistic regression was the primary estimator and required no hyperparameter tuning. For estimators requiring tuning, settings were selected from inner out-of-fold (OOF) predictions by maximizing standardized high-specificity partial AUROC (hs-

pAUC), with ties resolved by Brier score, deviation of the calibration slope from 1, and predefined grid order.

S3. Repeated nested cross-validation and statistical implementation

S3.1. Resampling and leakage control

Patient-level stratified resampling used an outer five-fold loop repeated 10 times and, within each outer training set, an inner five-fold loop repeated three times. Each patient contributed one outer held-out probability per repeat, yielding 5330 OOF records. Preprocessing based on the observed data, tuning, fitting, and operating point selection were restricted to the relevant training partition; outer held-out observations did not contribute to preprocessing, tuning, or threshold selection.

S3.2. Model fitting and operating-point selection

For the primary model, missing continuous values were replaced with medians from the training partition on the original measurement scale. CEA, CYFRA21-1, WBC, and lactate were transformed as log2(x + 1). The six continuous CSF variables and systemic therapy line were standardized using the training mean and population standard deviation; the brain metastasis indicator remained binary. The fitted estimator was conventional unpenalized logistic regression with an intercept. Median imputation was retained as the primary deterministic, deployment-compatible preprocessing rule; it was not assumed to be statistically superior to multiple imputation.

Each outer split received an operating point derived from inner-OOF predictions. All unique inner-OOF probabilities were considered, and sensitivity was maximized among thresholds with specificity strictly greater than 0.90; the lowest threshold resolved ties. The selected threshold was then applied to the corresponding outer held-out fold. Outer held-out specificity was not required to remain above 0.90.

S3.3. Performance aggregation and uncertainty estimation

Metrics included AUROC, standardized hs-pAUC over false positive rates 0–0.10, Brier score, log loss, calibration intercept and slope, sensitivity, specificity, predictive values where reported, and balanced accuracy. Each metric was calculated from the 533 held-out predictions in each outer repeat and then averaged across 10 repeats. Classification metrics used the threshold assigned to each split and attached to every corresponding OOF record.

Primary 95% confidence intervals used 5000 patient cluster bootstrap samples stratified by reference group. Sampling was performed separately within the 366 Any-LM and 167 BM-only groups, with all 10 OOF records for each sampled patient retained together. Metrics were calculated within each repeat and averaged before percentile intervals were obtained; thresholds were not re-estimated within bootstrap samples.

S3.4. Graphical summaries and descriptive full-cohort refit

Figure 3A, Figure 3C, and Figure 3D display each patient's mean of 10 OOF probabilities, whereas the annotated primary estimates are means calculated across repeats. Figure 4A pools all 5330 OOF probabilities for visualization; its model operating point reports the mean across repeats obtained with thresholds derived separately in the inner loop for each split. The 50 inner-derived thresholds had a median of 0.6038 (IQR, 0.5923–0.6248).

A separate descriptive model was refitted to all 533 patients using medians from the full cohort, the same transformations, and scaling by the population standard deviation. This refit supplied the probability equation, adjusted associations and pathway classifications. The fixed threshold of 0.6103 was derived by full development resampling using the same specificity-constrained rule. Outputs from the full cohort were not used to estimate outer held-out performance (Table S3; Figure S4).

S4. Sensitivity and exploratory analyses

S4.1. Missing-data and model-robustness analyses

The primary strategy used median imputation fitted within each training partition. As a formal sensitivity analysis, stochastic multiple imputation by chained equations used Bayesian-ridge conditional models with posterior sampling within every training partition (20 imputations; 10 iterative rounds). CEA, CYFRA21-1, WBC, and lactate were imputed on the log2(x + 1) scale. The reference diagnosis was excluded from the imputation model, assessment observations did not estimate imputation parameters, and preprocessing, model fitting, and operating-point selection were repeated within the saved nested cross-validation structure. Held-out predicted probabilities were averaged across the 20 imputations before evaluation and threshold application. A separate chained-equations sensitivity analysis generated one completed dataset within each training partition (m = 1) and remained a single-imputation analysis without Rubin pooling. Complete-case analysis used the 340 patients with all eight predictors observed and was supportive rather than directly comparable. Missing-data results used 5000 patient-cluster bootstrap samples (Figure S3; Table S5A).

Estimator sensitivity included Firth regression with FLIC intercept correction and the 12-estimator benchmark. Threshold sensitivity compared the strict primary rule with a training-derived 90th-percentile reference among BM-only inner-OOF probabilities.

S4.2. Routine-strategy and illustrative pathway-projection analyses

Routine strategies used the retained brain MRI and first CSF cytology results from the index diagnostic evaluation. First CSF cytology was the result from the first lumbar puncture during that episode. Unavailable MRI was counted as not identified by the pragmatic pathway, but not as a negative test or false negative. The combined strategy was positive when either routine test was positive. Routine strategies used unique patients; model classifications were calculated within each outer repeat and then averaged. Because both routine tests informed the documented integrated clinical diagnosis, comparisons were descriptive within the reference diagnosis cohort.

Intervals for routine strategies used 5000 patient bootstrap samples stratified by reference group. Intervals for the repeated model retained each patient's 10 OOF records as a cluster. Complementary OOF flagging was evaluated among Any-LM patients not identified by both routine strategies. The illustrative pathway projection used the descriptive threshold of 0.6103 from the full cohort. It counted routine-negative patients flagged for further LM-directed assessment, potential additional Any-LM identifications conditional on confirmation, and BM-only patients flagged for workup. Rates per 100 used all 533 patients and were rounded independently. The scenario did not measure diagnostic behavior, treatment change, outcomes, or clinical benefit (Table S4).

S4.3. Lactate-focused analyses

Lactate analyses were exploratory. The adjusted dose–response analysis used 510 patients with observed lactate and restricted cubic splines at the 5th, 35th, 65th, and 95th percentiles (1.40, 1.91, 2.99, and 6.35 mmol/L). Other predictors used preprocessing parameters from the full cohort. This available case analysis was not an OOF performance estimate. Likelihood ratio tests assessed overall contribution and nonlinearity, and no lactate cutoff was estimated.

Paired ablation analyses compared the eight-factor model with models omitting each predictor while holding folds, preprocessing, estimator, and threshold algorithm constant. Percentile intervals used 2000 patient cluster bootstrap samples stratified by reference group, retaining all 10 predictions per patient. Within Any-LM, observed pair partial Spearman correlations related lactate to seven CSF measures after adjustment for brain metastasis burden and therapy line. Percentile intervals were based on 2000 patient bootstraps, and Benjamini–Hochberg adjustment covered the seven nominal P values. These analyses were hypothesis generating and did not establish causality (Figure S5; Table S6).

Supplementary Figures

Figure S1. Study population flow

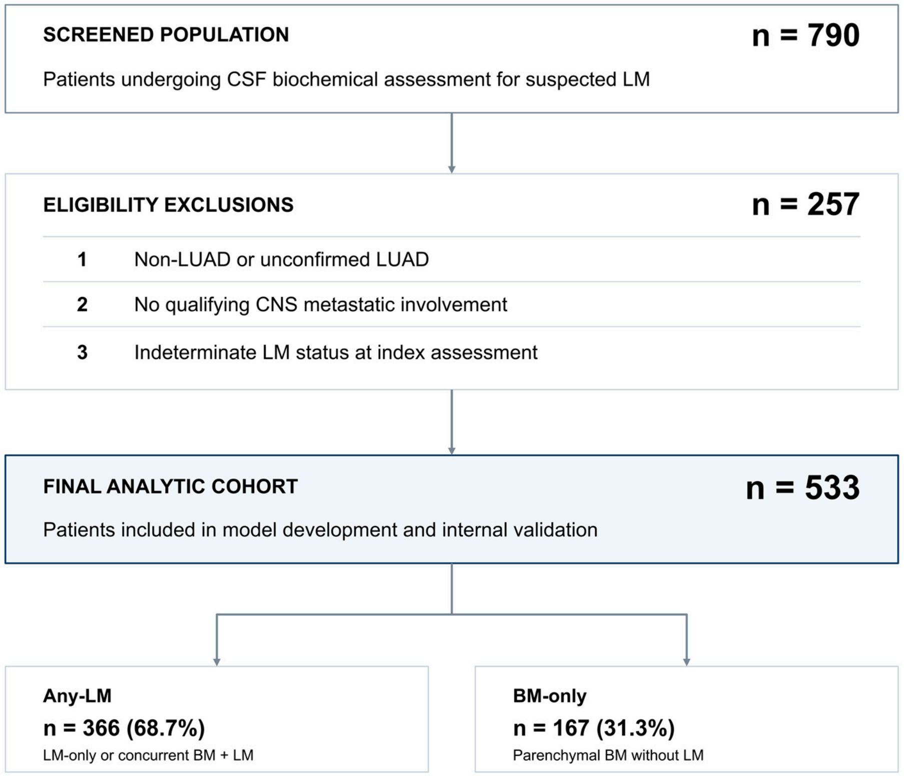

Counts are patient-level. CSF, cerebrospinal fluid; CNS, central nervous system; LUAD, lung adenocarcinoma; LM, leptomeningeal metastasis; BM, brain metastasis.

Figure S1. Of 790 screened records, 257 were excluded within three verified categories. Reason-specific exclusion counts were unavailable in the final analytic source. The final analytic cohort included 533 patients (Any-LM, n = 366; BM-only, n = 167).

Figure S2. Missing-data structure among the final eight predictors

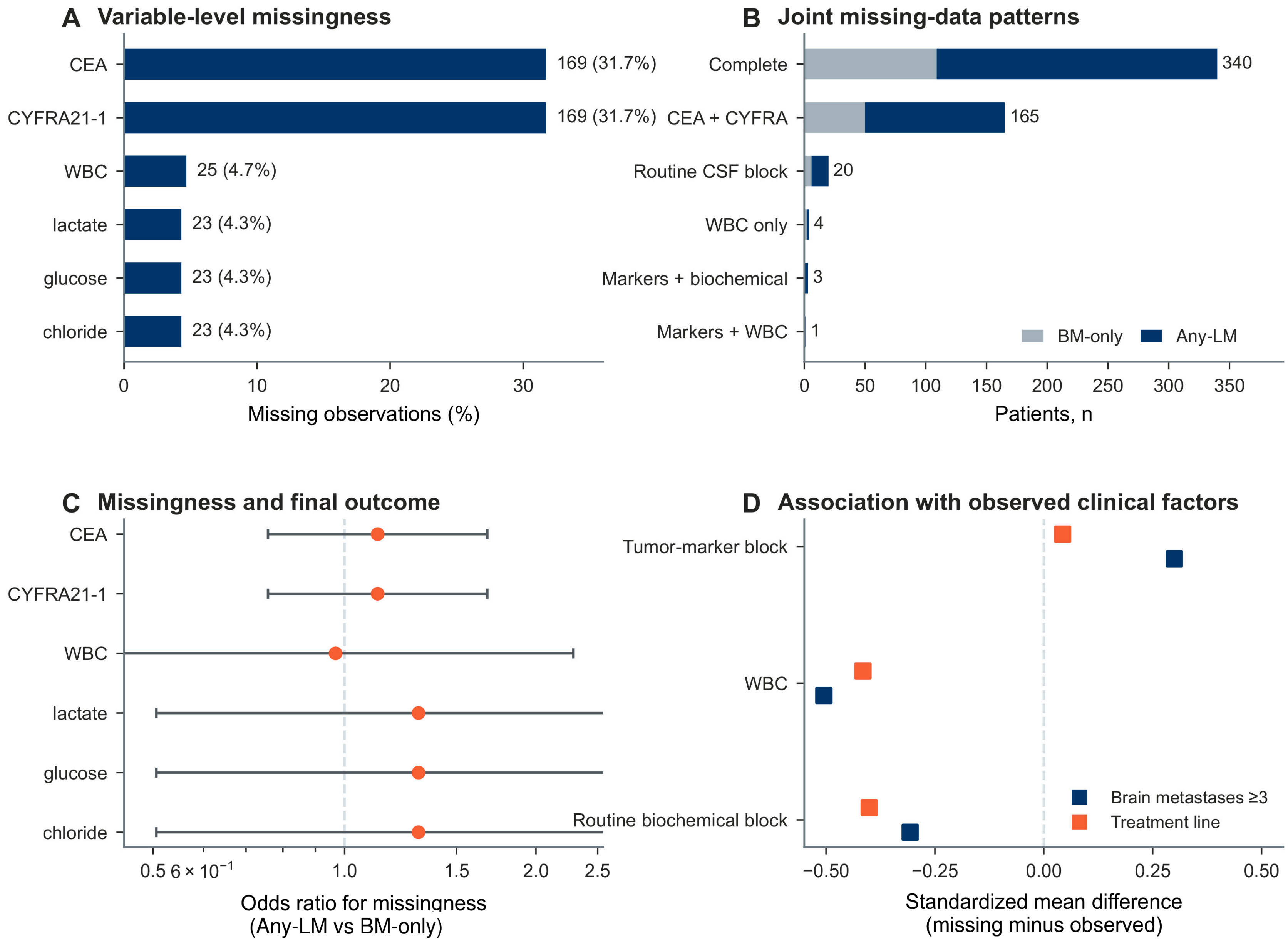

Figure S2. Pre-imputation availability and joint and group-specific missingness for the final eight predictors in the 533-patient analytic cohort.

Figure S3. Sensitivity of model performance to missing-data handling

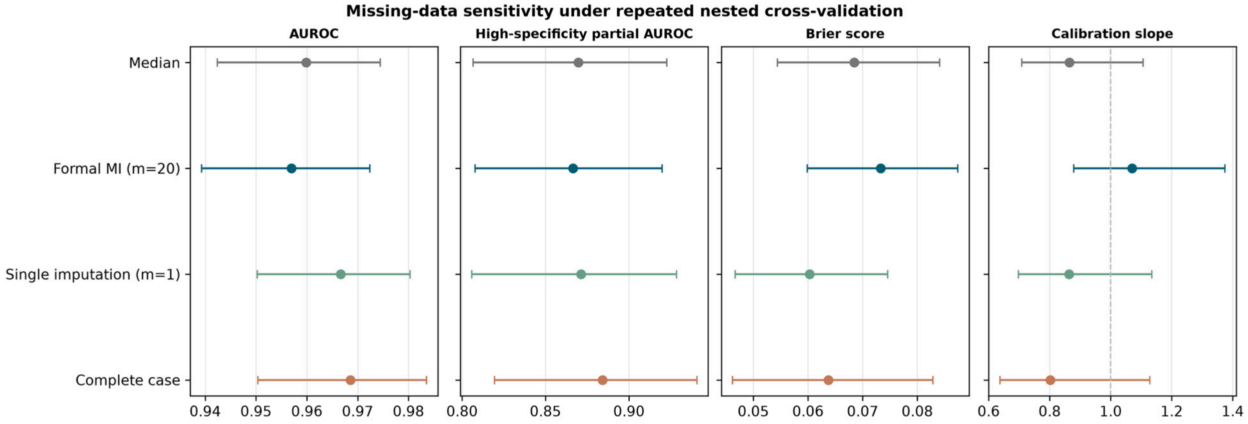

Figure S3. Repeated nested cross-validation performance under fold-local median imputation (primary; n = 533), formal fold-local multiple imputation (m = 20; n = 533), fold-local chained-equations single imputation (m = 1; n = 533), and complete-case analysis (n = 340). Formal multiple-imputation probabilities were averaged across imputations for each held-out patient before evaluation.

Figure S4. Stability of repeated nested cross-validation

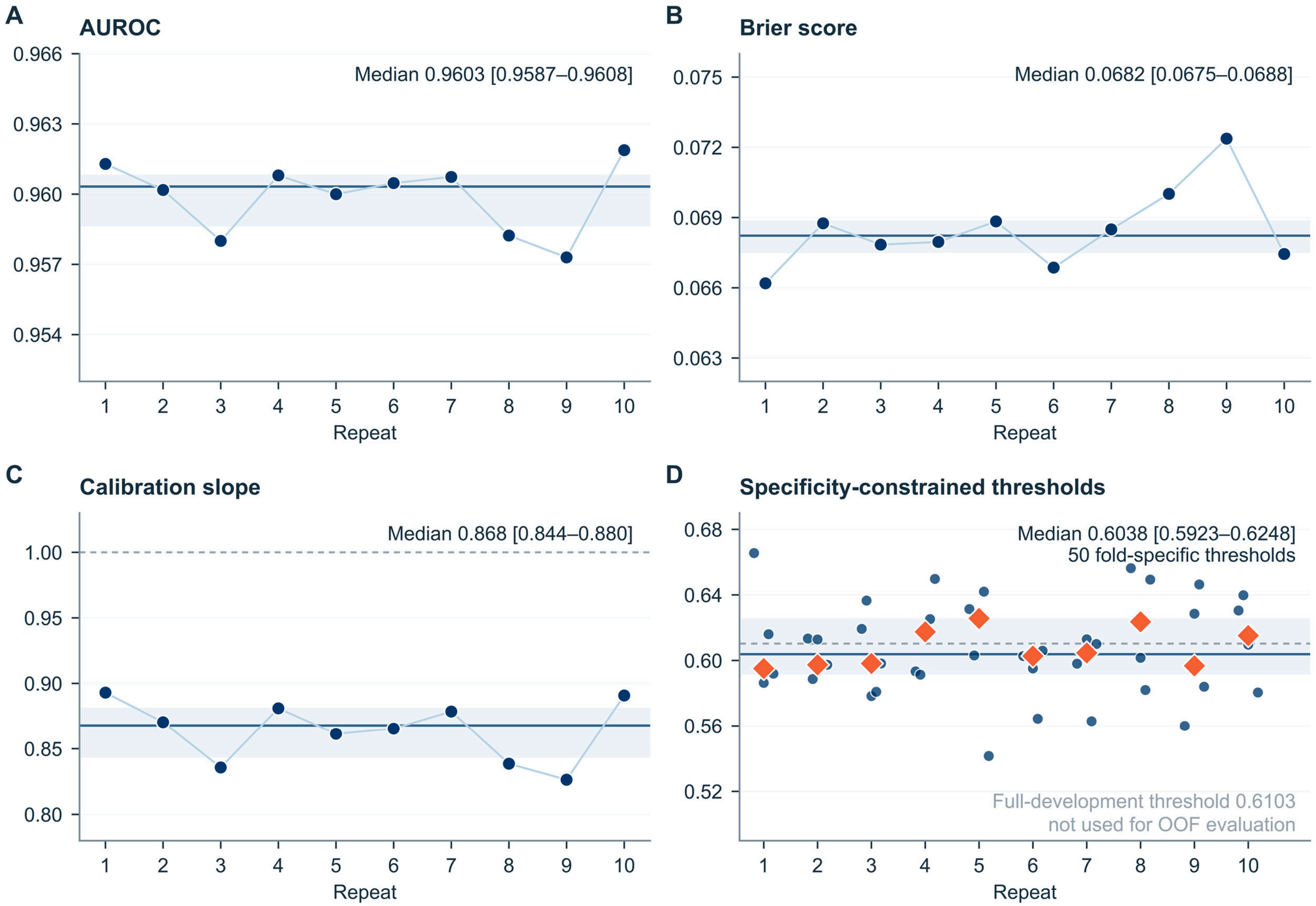

Figure S4. Performance across repeats and the distribution of 50 operating thresholds derived in the inner loop. The median inner-derived threshold was 0.6038 (IQR, 0.5923–0.6248); the descriptive full cohort threshold of 0.6103 was not an OOF threshold.

Figure S5. Exploratory lactate-focused analyses

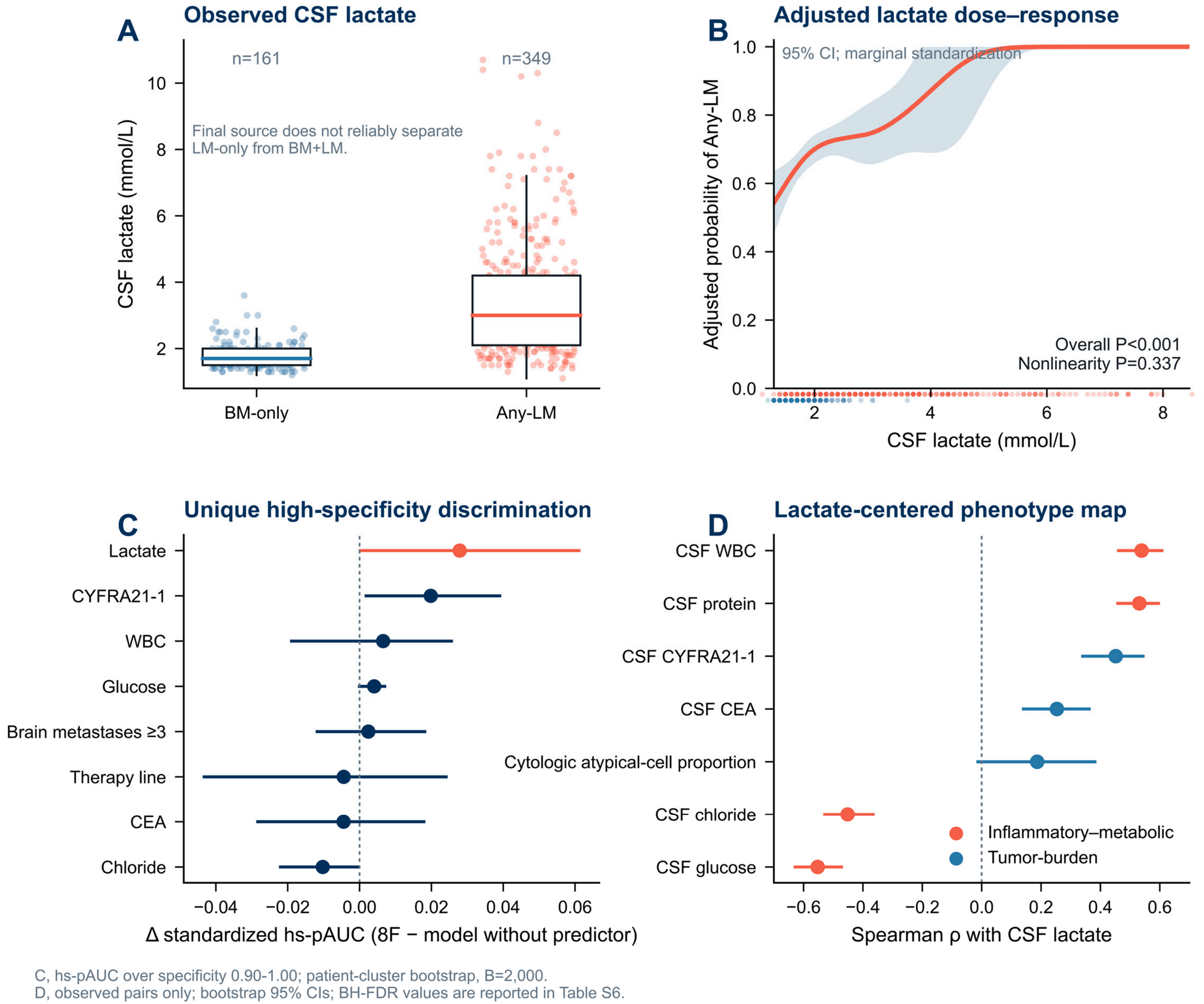

Figure S5. (A) Observed CSF lactate by reference group; (B) adjusted spline-based dose–response; (C) paired predictor ablation differences in standardized high-specificity partial AUROC; and (D) observed pair partial rank correlations within Any-LM. All lactate analyses were exploratory.

Supplementary Tables

Table S1. Definitions, availability, and preprocessing of all candidate predictors

Variable	Operational definition	Unit	Available; missing	Predictor-specific coding or transformation	Final model
Age, years	Chronological age during routine diagnostic evaluation.	years	533/533; 0 (0.0%)	Identity	No
Female sex	Sex coded 1=female and 0=male.	Binary	533/533; 0 (0.0%)	Binary 0/1; male reference	No
No documented smoking history	Never smoker or no documented smoking history versus ever smoker.	Binary	533/533; 0 (0.0%)	Binary 0/1; ever-smoker reference	No
Driver gene status	Five-level driver-alteration category; reference=no known driver or not tested.	Categoric al	533/533; 0 (0.0%)	One-hot encoded	No
Line of systemic therapy	Number of systemic-therapy lines; unrecorded values were coded as 0 according to the prespecified coding rules.	line count	533/533 after coding; raw unrecorded n unavailable	Identity	Yes
Time since lung adenocarcinoma diagnosis	Months from lung adenocarcinoma diagnosis to routine diagnostic evaluation.	months	533/533; 0 (0.0%)	Identity	No
Primary lung lesion radical resection	Prior radical resection of the primary lung lesion: yes versus no/unknown.	Binary	533/533; 0 (0.0%)	Binary; no/unknown reference	No
Primary CNS metastasis	CNS metastasis present at initial cancer presentation: yes versus no/unknown.	Binary	533/533; 0 (0.0%)	Binary; no/unknown reference	No
Prior brain radiotherapy	Brain radiotherapy before routine diagnostic evaluation: yes versus no/unknown.	Binary	533/533; 0 (0.0%)	Binary; no/unknown reference	No
Brain metastases ≥3	Imaging-derived burden: at least 3 versus fewer than 3 or undocumented.	Binary	533/533 after coding; raw undocumented n unavailable	Binary; <3/undocumented reference	Yes
CSF CEA	CEA concentration in the retained CSF record.	ng/mL	364/533; 169 (31.7%)	log2(x + 1)	Yes
CSF CYFRA21-1	CYFRA21-1 concentration in the retained CSF record.	ng/mL	364/533; 169 (31.7%)	log2(x + 1)	Yes
CSF NSE	NSE concentration in the retained CSF record.	ng/mL	364/533; 169 (31.7%)	log2(x + 1)	No
CSF SCC	SCC concentration in the retained CSF record.	ng/mL	364/533; 169 (31.7%)	log2(x + 1)	No
CSF ProGRP	ProGRP concentration in the retained CSF record.	pg/mL	364/533; 169 (31.7%)	log2(x + 1)	No
CSF WBC	White blood cell count in the retained CSF record.	cells/µL	508/533; 25 (4.7%)	log2(x + 1)	Yes
CSF total cell count	Total cell count in the retained CSF record.	cells/µL	508/533; 25 (4.7%)	log2(x + 1)	No
CSF differential composition (PMN%)	Polymorphonuclear-cell percentage; PMN and MN were exact complements and represented one information unit.	%	469/533; 64 (12.0%)	Original percentage scale	No

Variable	Operational definition	Unit	Available; missing	Predictor-specific coding or transformation	Final model
CSF glucose	Glucose concentration in the retained CSF record.	mmol/L	510/533; 23 (4.3%)	Identity	Yes
CSF protein	Total protein concentration in the retained CSF record.	mg/dL	510/533; 23 (4.3%)	log2(x + 1)	No
CSF lactate	Lactate concentration in the retained CSF record.	mmol/L	510/533; 23 (4.3%)	log2(x + 1)	Yes
CSF chloride	Chloride concentration in the retained CSF record.	mmol/L	510/533; 23 (4.3%)	Identity	Yes
CSF ADA	Adenosine deaminase activity in the retained CSF record.	U/L	510/533; 23 (4.3%)	log2(x + 1)	No

Availability and missingness are reported before imputation. Except where stated, continuous predictors were imputed using the median from each training partition and standardized with parameters from that partition; Table S1 shows only predictor-specific transformations. Systemic therapy line and brain metastasis burden were complete only after application of the prespecified coding rules, and the original numbers of unrecorded values could not be recovered. PMN, polymorphonuclear; MN, mononuclear; ADA, adenosine deaminase; CEA, carcinoembryonic antigen; NSE, neuron-specific enolase; SCC, squamous cell carcinoma antigen; ProGRP, pro-gastrin-releasing peptide.

Table S2. Predictor-set development and estimator benchmark

A. Candidate predictor specifications evaluated during model development

Configuration	Predictors, n	Included predictors
Full 23	23	All 23 nonredundant information units in Table S1
10F	10	Eight-factor core plus CSF protein and CSF ProGRP
9F	9	Eight-factor core plus CSF ProGRP
8F final	8	CSF CEA; CSF CYFRA21-1; CSF WBC; CSF lactate; CSF glucose; CSF chloride; brain metastases ≥3; systemic-therapy line
7F	7	Eight-factor model without brain-metastasis burden
6F	6	Six CSF predictors in the eight-factor model

Developmental stability sets D1–D3 were generated within the original development splits and informed the compact configurations summarized below; they were not submitted as separate fixed predictor lists.

B. Estimator families and inner-loop tuning dimensions

Estimator	Implementation (version)	Parameters tuned in the inner loop
Conventional logistic	scikit-learn 1.9.0	None
Firth/FLIC	R logistf 1.26.1; R 4.6.0	None
Ridge	scikit-learn 1.9.0	Penalty strength
Lasso	scikit-learn 1.9.0	Penalty strength
Elastic net	scikit-learn 1.9.0	Penalty strength; L1 ratio
RCS/GAM	R mgcv; version not recorded	None; fixed spline specification
Random forest	scikit-learn 1.9.0	Feature sampling; minimum leaf size
ExtraTrees	scikit-learn 1.9.0	Feature sampling; minimum leaf size
HistGradientBoosting	scikit-learn 1.9.0	Learning rate; L2 regularization; leaf nodes
XGBoost	xgboost 3.3.0	Learning rate; depth; child weight; subsampling
LightGBM	lightgbm 4.6.0	Learning rate; child samples; leaves; L2 regularization
RBF-SVM	scikit-learn 1.9.0	Penalty strength; kernel scale

All estimators used the same eight predictors, with preprocessing fitted within each fold. Tuned estimators were selected within each outer training set using inner OOF hs-pAUC; ties were resolved by Brier score, deviation of the calibration slope from 1, and predefined grid order. The operating point was derived separately for each outer split. Only the tuned parameter dimensions are shown. Python 3.12.13; NumPy 2.5.1; pandas 3.0.3; scikit-learn 1.9.0; XGBoost 3.3.0; LightGBM 4.6.0; R 4.6.0; logistf 1.26.1. The contemporaneous mgcv version was not recorded.

Table S3. Final full-cohort model coefficients and probability equation

Predictor	Unit	Transformation	Full-cohort median; center; scale	Beta	OR	95% CI
Intercept	-	-	-	2.641310	-	-
CSF CEA	ng/mL	log2(x + 1)	9.16; 3.632207; 2.587742	1.306179	3.692	2.018-6.755
CSF CYFRA21-1	ng/mL	log2(x + 1)	2.38; 2.226985; 1.688140	2.249513	9.483	3.244-27.719
CSF WBC	cells/μL	log2(x + 1)	8; 3.249089; 1.431417	1.080457	2.946	1.685-5.151
CSF lactate	mmol/L	log2(x + 1)	2.3; 1.865344; 0.506055	1.809515	6.107	2.535-14.717
CSF glucose	mmol/L	Identity	3.36; 3.364878; 1.328049	-0.408492	0.665	0.416-1.063
CSF chloride	mmol/L	Identity	124; 123.607880; 4.710980	-0.447462	0.639	0.366-1.118
Brain metastases ≥3	Binary	0/1	Reference: <3/undocumented; not standardized	1.349649	3.856	1.814-8.196
Line of systemic therapy	line count	Identity	2; 1.930582; 1.747918	0.867122	2.380	1.530-3.703

P(Any-LM) = 1 / [1 + exp(-eta)], where eta = beta0 + beta1Z1 + ... + beta8Z8 and each Z is defined in Table S3. Missing continuous values were replaced by the listed raw median before transformation; transformed values were then centered and scaled as shown. Values are rounded to six decimals. ORs for continuous predictors are per 1 SD increase on the model scale. The fixed threshold of 0.6103 was used only for descriptive pathway analyses in the full cohort, not for estimating outer held-out performance.

Table S4. Strategy-level performance, complementary flagging, and illustrative pathway projection

A. Diagnostic classifications and performance by strategy

Strategy	Any-LM identified / strategy-level rate	BM-only not flagged / BM-only non-flagging rate	PPV	NPV	Balanced accuracy
Brain MRI strategy	196/366 (53.6%; 48.4-58.7)	137/167 (82.0%; 76.0-88.0)	86.7% (82.8-90.6)	44.6% (41.6-47.9)	67.8% (63.8-71.8)
First CSF cytology	301/366 (82.2%; 78.1-86.1)	167/167 (100.0%; 97.8-100.0)	100.0% (98.8-100.0)	72.0% (67.6-76.6)	91.1% (89.1-93.0)
Brain MRI-or-first CSF cytology	325/366 (88.8%; 85.2-91.8)	137/167 (82.0%; 76.0-88.0)	91.5% (89.0-94.0)	77.0% (71.9-82.4)	85.4% (82.0-88.7)
Eight-factor model, repeated OOF	333.8 mean/repeat (91.2%; 88.3-93.8)	150.5 mean/repeat (90.1%; 85.7-93.9)	95.3% (93.4-97.1)	82.4% (77.9-86.9)	90.7% (88.0-93.0)

Values in parentheses are 95% confidence intervals. Routine strategies used unique patients. Brain MRI was evaluable in 510 patients; the 23 examinations that were unavailable or not performed were counted as not identified by the pragmatic MRI strategy, not as MRI test false negatives. The MRI percentage is therefore an Any-LM identification rate for the full cohort rather than sensitivity among patients with an evaluable test. The BM-only non-flagging rate was calculated as the proportion of all BM-only patients not flagged by the pragmatic strategy, including patients without an evaluable MRI examination. Repeated OOF model counts are means per outer cross-validation repeat. All other intervals were estimated from 5000 patient-level bootstrap resamples; for OOF estimates, all 10 records per sampled patient were retained. For the BM-only non-flagging rate and PPV of first CSF cytology, no failures were observed, and two-sided exact Clopper-Pearson intervals were reported because nonparametric bootstrap intervals degenerate at 100%. Comparisons are descriptive because MRI and cytology informed the reference diagnosis.

B. Complementary identification and illustrative pathway projection

Analysis	Count	Rate	Interpretation
Any-LM not identified by MRI strategy and flagged by the full-cohort model	157/170	92.4%	Descriptive complementarity at threshold 0.6103
Any-LM not identified by first CSF cytology and flagged by the full-cohort model	59/65	90.8%	Descriptive complementarity at threshold 0.6103
Any-LM not identified by either routine strategy	41	-	Unique-patient OOF denominator
Repeated OOF model positive among the 41 double-negative Any-LM patients	37.6 mean/repeat	91.7% (83.5-98.2)	Flagged for further LM-directed assessment
Projected model-guided further assessment	51	9.6 per 100	Full-cohort scenario
Projected potential additional Any-LM identification	38	7.1 per 100	Requires subsequent diagnostic confirmation
Projected BM-only workup burden	13	2.4 per 100	Additional assessment burden

The first two rows use the descriptive refit in the full cohort at threshold 0.6103; the MRI and cytology groups overlap. The double-negative OOF analysis used thresholds derived separately in the inner loop for each split. Projected values per 100 use the observed cohort prevalence, and separately rounded components may not sum to the total. Model positivity indicates further LM-directed assessment, not diagnostic confirmation or demonstrated clinical benefit.

Table S5. Sensitivity analyses

A. Sensitivity of conventional logistic-model performance to missing-data handling

Missing-data strategy	N	AUROC	hs-pAUC	Brier score	Log loss	Calibration intercept	Calibration slope
Fold-local median imputation (primary)	533	0.960 (0.942-0.974)	0.870 (0.807-0.923)	0.068 (0.054-0.084)	0.238 (0.189-0.295)	0.007 (-0.243 to 0.261)	0.864 (0.708-1.106)
Formal fold-local multiple imputation (m = 20)	533	0.957 (0.939 to 0.972)	0.866 (0.808 to 0.920)	0.073 (0.060 to 0.087)	0.246 (0.204 to 0.292)	-0.017 (-0.252 to 0.230)	1.070 (0.879 to 1.375)
Fold-local chained-equations single imputation (m = 1)	533	0.967 (0.950-0.980)	0.871 (0.806-0.928)	0.060 (0.047-0.075)	0.222 (0.169-0.283)	-0.008 (-0.256 to 0.288)	0.864 (0.697-1.134)
Complete-case analysis	340	0.969 (0.950-0.984)	0.884 (0.820-0.941)	0.064 (0.046-0.083)	0.223 (0.159-0.297)	-0.049 (-0.374 to 0.388)	0.802 (0.637-1.128)

Values are repeated outer-OOF point estimates with 95% confidence intervals from 5000 outcome-stratified patient-cluster bootstrap samples. Median imputation was retained as the primary deterministic, deployment-compatible preprocessing rule. Formal multiple imputation used 20 stochastic completed datasets fitted separately within every training partition; held-out probabilities were averaged before evaluation. The chained-equations sensitivity analysis used one completed dataset per training partition and remains labeled single imputation. Complete-case analysis used a smaller selected cohort and is supportive rather than directly comparable. High-specificity partial AUROC is standardized over false-positive rates 0–0.10. Compared with median imputation, formal multiple imputation changed AUROC by −0.0030 (95% CI, −0.0066 to 0.0006), Brier score by +0.0048 (0.0010–0.0085), sensitivity by −0.020 (−0.033 to −0.008), and specificity by −0.002 (−0.020 to 0.015).

B. Estimator and threshold sensitivity analyses

Sensitivity analysis	Cohort	Operating basis	Outer held-out sensitivity	Outer held-out specificity
Conventional logistic primary	N=533	Fold-specific inner specificity >0.90	91.2% (88.3-93.8)	90.1% (85.7-93.9)
Firth/FLIC	N=533	Fold-specific inner specificity >0.90	91.2% (88.3-93.8)	90.1% (85.7-93.8)
Alternative P90 threshold	N=533	Training-derived BM-only 90th percentile	91.3%	89.9%

Rows for conventional logistic regression and Firth/FLIC show patient cluster bootstrap 95% CIs. The alternative threshold row is a supportive point estimate and is not directly interchangeable with the strict primary operating point. Missing data analyses are reported only in Table S5A and are not duplicated here.

Table S6. Lactate-centered partial-rank-correlation results in observed Any-LM pairs

Variable	Observed-pair n	Partial Spearman ρ	95% CI	Nominal P	BH-FDR q
CSF WBC	346	0.539	0.455-0.612	<0.001	<0.001
CSF protein	349	0.531	0.457-0.601	<0.001	<0.001
CSF CYFRA21-1	233	0.446	0.329-0.548	<0.001	<0.001
CSF CEA	233	0.246	0.126-0.360	<0.001	<0.001
Cytologic atypical-cell proportion	98	0.166	-0.042 to 0.363	0.106	0.106
CSF chloride	349	-0.446	-0.531 to -0.353	<0.001	<0.001
CSF glucose	349	-0.550	-0.631 to -0.464	<0.001	<0.001

Note: Observed pairs only. Partial Spearman correlations were adjusted for brain metastasis burden (≥3 vs. fewer/undocumented) and systemic therapy line. The 95% CIs are percentile intervals from 2000 patient-level bootstrap samples. Two-sided nominal P values used the conventional t approximation for residualized rank partial correlation with two adjustment variables (df = n − 4); Benjamini–Hochberg q values controlled the false discovery rate across seven correlations. These analyses were exploratory.
